# Innate immune activation and aberrant function in the R6/2 mouse model and Huntington’s disease iPSC-derived microglia

**DOI:** 10.3389/fnmol.2023.1191324

**Published:** 2023-06-20

**Authors:** Julien Gasser, Gaelle Gillet, Jorge S. Valadas, Laura Rouvière, Apoorva Kotian, Wenqiang Fan, James Keaney, Irena Kadiu

**Affiliations:** ^1^Neuroinflammation Focus Area, Neuroscience Research, UCB Biopharma SRL, Braine-l’Alleud, Belgium; ^2^Development Science, UCB Biopharma SRL, Slough, United Kingdom

**Keywords:** microglia, phagocytosis, migration, neuroinflammation, Huntington’s disease, dendritic spines, R6/2 model, cyTOF

## Abstract

Huntington’s disease (HD) is an inherited autosomal dominant neurodegenerative disease caused by CAG repeats in exon 1 of the *HTT* gene. A hallmark of HD along with other psychiatric and neurodegenerative diseases is alteration in the neuronal circuitry and synaptic loss. Microglia and peripheral innate immune activation have been reported in pre-symptomatic HD patients; however, what “activation” signifies for microglial and immune function in HD and how it impacts synaptic health remains unclear. In this study we sought to fill these gaps by capturing immune phenotypes and functional activation states of microglia and peripheral immunity in the R6/2 model of HD at pre-symptomatic, symptomatic and end stages of disease. These included characterizations of microglial phenotypes at single cell resolution, morphology, aberrant functions such as surveillance and phagocytosis and their impact on synaptic loss *in vitro* and *ex vivo* in R6/2 mouse brain tissue slices. To further understand how relevant the observed aberrant microglial behaviors are to human disease, transcriptomic analysis was performed using HD patient nuclear sequencing data and functional assessments were conducted using induced pluripotent stem cell (iPSC)-derived microglia. Our results show temporal changes in brain infiltration of peripheral lymphoid and myeloid cells, increases in microglial activation markers and phagocytic functions at the pre-symptomatic stages of disease. Increases in microglial surveillance and synaptic uptake parallel significant reduction of spine density in R6/2 mice. These findings were mirrored by an upregulation of gene signatures in the endocytic and migratory pathways in disease-associated microglial subsets in human HD brains, as well as increased phagocytic and migratory functions of iPSC-derived HD microglia. These results collectively suggest that targeting key and specific microglial functions related to synaptic surveillance and pruning may be therapeutically beneficial in attenuating cognitive decline and psychiatric aspects of HD.

## Introduction

Huntington’s disease (HD) is a fatal neurodegenerative disorder caused by a polyglutamine (polyQ) repeat expansion in the *HTT* gene. It encodes for mutant Huntingtin (mHTT), a ubiquitous protein with an abnormally long PolyQ stretch, contributing to systemic and central nervous system (CNS) toxicities through gain-of-toxic function interference with normal protein folding, induced oxidative stress, and programmed cell death.

Peripheral widespread systemic toxicity and mHTT pathology has been reported in blood leukocytes, liver, pancreas, kidney and cardiac cells among others (Sassone et al., 2009; van der Burg et al., 2009). Human leukocytes express high levels of mHTT (Moscovitch-Lopatin et al., 2010; Weiss et al., 2012). These cells display activated phenotypes characterized by increased pro-inflammatory cytokines (TNF, IL1β, IL6, IL8, etc.) and lowered tissue-trophic factors such as transforming growth factor-β1 (TGF-β1) in the serum or exaggerated pro-inflammatory response to lipopolysaccharide (LPS) in cell culture (Schure et al., 2010; Di Pardo et al., 2013). Increased leukocyte activation and increased brain infiltration of NFκB-active monocytes has been reported as early as pre-symptomatic stages of disease and correlates with rates of disease progression (Di Pardo et al., 2013). In animal models of HD including R6/2 mice, peripheral innate immunity has been shown to be dysregulated; however, it remains unclear due to contradicting reports what functions are impacted and their involvement in murine mHTT pathobiology (Kwan et al., 2012; Träger et al., 2015; Lee et al., 2018).

In the CNS, mHTT toxicity is displayed in the form of selective vulnerability of medium spiny neurons (MSNs) and corticostriatal projection neurons (CPNs) modulating movement and cognition, respectively. Selective loss of MSNs and CPNs leads to progressive degeneration of basal ganglia and cortex manifesting as cognitive, behavioral, and motor disturbances including memory loss, depression and choreic (involuntary) movement. Although mHTT is the primary disease trigger in HD, the progressive loss of neuronal subtypes is thought to be non-cell autonomous. Neighboring cells such as microglia, essential for optimal neuronal/circuitry function and survival, are thought to contribute to the non-cell autonomous degeneration mechanisms in HD through increased release of pro-inflammatory and oxidative stress factors and maladaptive synaptic pruning. Imaging studies have established a link between synaptic density and cognitive performance across a range of neurodegenerative disease characterized by cognitive decline (Delva et al., 2022; Mecca et al., 2022). In HD in particular, imaging studies utilizing ^11^C-UCB-J, a radioligand targeting synaptic vesicle protein 2A (SV2A), have shown the degree of synaptic loss (starting in the striatum) associates with earlier onset of cognitive and motor symptoms (Delva et al., 2022). Additionally, increased signal of ^11^C-(R)-PK11195, a positron emission tomography (PET) radioligand of microglial/glial translocator protein (TSPO) in striatum and cortex correlated with dopamine receptor loss and could predict disease onset and clinical severity (Tai et al., 2007; Politis et al., 2011). Autonomous microglial activation has been reported in various rodent models of HD including R6/2 characterized by increased Iba1 staining in regions of brain pathology (Crotti et al., 2014); However, what Iba1+ increases and microglial activation signifies in terms of functional impact on disease pathobiology in HD remains unclear.

Currently, only symptomatic treatments are available. These temporarily address some of the HD disease symptoms without impacting the underlying disease pathobiology or progressive degeneration. Therefore, a thorough understanding of the non-cell autonomous mechanistic drivers of disease progression in human disease and interrogating their presence in rodent models among other tools for testing of therapeutic hypotheses is critical. More specifically, understanding what microglial functions are dysregulated in disease and their impact on neuronal health is the first step in identification of therapeutic targets that will potentially correct the underlying problem.

Our study addresses some of these knowledge gaps in HD human microglial functional pathobiology through comprehensive imaging, transcriptomic, phenotypic, and functional interrogation of human HD tissues/cells, and the R6/2 mouse model. It provides novel insights into disrupted innate immune biologies in HD as potential hypothesis entry points for testing of neuroimmune modulators as future therapies for HD.

## Materials and methods

### Animals

R6/2 transgenic mice (B6CBA-Tg (HDexon1)62Gpb/3 J, Jackson Labs # 006494) and non-transgenic litter mates (ntg) were in-licensed from King’s College London. Mice were housed in ventilated cages with nesting cups (Kraft paper, CARFIL QUALITY, Beyntellus 3) on a 12/12 h light/dark cycle with lights on at 06:00 h and had al libitum access to rodent chow (Safe, Plant diets & Custom diet, Route de Saint Bris – 89,290 Augy – France) and tap water. The temperature in the husbandry was maintained at 20–22°C and humidity at about 40%. Following a habituation period of a minimum 1 week, mice were euthanized, and tissues collected at 7, 9, 10, 13, and 15 weeks of age for *ex vivo*, *in vitro* functional and biochemical assays. All experimental procedures and humane endpoints were carefully reviewed and approved by a local Animal Experimentation and Well-Being Ethical Committee compliant with national legislation guidelines (Belgian Royal Decree regarding the protection of laboratory animals of May 29th, 2013) and the European directive (2010/63/EU). Additionally, all animal experiments were carried out in an AAALAC accredited facility.

### Human iPSC cell lines and tissues

Post-mortem frozen human brain tissues from the pre-central gyrus and the middle frontal gyrus of HD cases and non-demented controls were obtained from the Netherlands Brain Bank, Netherlands Institute for Neuroscience, Amsterdam (open access^1^). All Material has been collected from donors from whom written informed consent for brain autopsy and the use of the material and clinical information for research purposes had been obtained by the NBB. An HD patient iPSCs line heterozygous for pathologic CAG repeats in the HTT gene (one allele within the normal range, ~18 repeats, and the other allele ~40 repeats) were obtained under informed consent from European Bank for induced pluripotent Stem Cells (EBiSC, RCi004-A). These lines showed the presence of Pluripotency markers (e.g., SSEA4, TRA-1-81, OCT4, and SOX2) and ability to differentiate to endo-, meso-and ectodermal cell types. The isogenic control line with 21 CAG repeats on both alleles of the HTT gene was generated at Bioneer. Human brain samples negative for prion disease and iPSC lines that tested negative for infectious pathogens were cleared for use by the Ethics Committee of UCB Biopharma and registered by the UCB Biobank in compliance with applicable local legislature (Belgian Royal Decree on Biobanks of January 9th, 2018).

### Mouse bone marrow derived macrophage (BMDM) cell culture

WT and R6/2 litter mates were euthanized using a lethal dose of isoflurane followed by cervical dislocation. BMDM were isolated from femurs, tibias, humerus, and radius of 6 R6/2 and 6 WT mice flushing with 1x HBSS using a syringe with a 30-gauge needle. Flushed bone morrow was strained through a 40 μm filter (Falcon, 352340) followed by centrifugation at 300 xg for 10 min. The pellet was resuspended in 1 mL of DMEM-Glutamax supplemented with 20 ng/mL macrophage colony-stimulating factor (M-CSF), 1% penicillin–streptomycin 10,000 U/mL and 10% endotoxin-low heat-inactivated FBS. Cell numbers and viability were assessed using the Countess II automated cell counter (ThermoFisher Scientific). Monocytes in the bone marrow mix were allowed to adhere and differentiate in culture at 37°C, 5% CO_2_, for 7 days prior to use in functional assays. Floating non-monocytic cells were removed from the cultures through complete media exchanges every 2 days.

### Generation of pluripotent stem cell-derived microglia (iMGL)

iPSC HD and isogenic control lines were maintained in 6-well plates in feeder free conditions in complete mTESR plus medium (Stem Cell Technologies) for at least 2 passages. Two days before the start of the differentiation, iPSCs were cultured in complete Essential 8 Flex medium (ThermoFisher Scientific). Cells were fed fresh media daily, passaged every 4–5 days and maintained in a humidified incubator (20% O_2_, 5% CO2, 37°C).

The differentiation of iPSC to iMGL was performed as described previously in Abud et al. (2017) with some modifications. Briefly, on day-1, iPSCs were dissociated to single cells and plated in complete Essential 8 Flex medium (with 10 μM Y-27632 dihydrochloride, Abcam) at 0.75 × 10^6^ iPSCs per well in AggreWell^™^ 400 in 6-well plates pre-treated with Anti-Adherence Solution (Stem Cell Technologies). On day 0, the embryoid bodies were ready for iHPC generation and were cultured in T225 culture flasks (pre-treated with Anti-Adherence Solution from Stem Cell Technologies). iPSCs were first differentiated in induced Hematopoietic progenitor cells (iHPC) for 15 days. iHPC were collected by filtration through a 37 μm reversible cell strainer, centrifuged and resuspended in CryoStor^®^ CS10 (Stemcell Technologies) and stored in liquid nitrogen. Frozen iHPC were thawed in iHPC medium and then differentiated to iMGL for 14 days with iMGL Differentiation base medium, as described in Abud et al. (2017).

### BMDM and iMGL migration assay

BMDMs were seeded at a density of 50,000 cells/insert in 2-well culture insert 24-well plates (Ibidi). Cells were incubated at 37°C in 5% CO_2_ for 24 h. Culture inserts were then carefully removed followed leaving an unpopulated “scratched” area and the cell monolayer with fresh complete medium and imaging of the scratch area using an EVOS digital inverted light microscope. Extent of microglia cell migration into the scratch area was quantified using a customized ImageJ script.

iMGL cultured for 30 days were replated in a clear bottom 96-well plate (ImageLock) coated with poly-D-lysine (1 mg/mL) at 40,000 cells per well and were allowed to rest at 37°C in 5% CO_2_ for 1 h. Cells were then mechanically removed from the center of the well with a Incucyte^®^ 96-Well Woundmaker Tool. Migration of iMGL to the generated wound was measured in images acquired in Incucyte S3 (Sartorius) using the Relative Wound Density metric (percentage of cells that migrated to the wound compared to the cells that remained in the unscratched area).

### Assessment of phagocytic activity in murine BMDM and human iMGL cell cultures

To assess phagocytic function in BMDM from transgenic and litter mate control mice, cells were plated at a density of 30,000 cells/well in 96-well poly-D-lysine-coated plates, and allowed to rest at 37°C, and 5% CO_2_ overnight. Cells were then exposed to pHrodo Red Zymosan Bioparticles^™^ (12.5 μg/mL per well; ThermoFisher Scientific) for 1 h at 37°C in 5% CO_2_. Cells were washed once with 1X PBS for 5 min and fixed in 4% PFA for 10 min at RT. PFA was removed and fixed cells were washed twice with 1X PBS. Fixed cells were stained with either HCS CellMask Blue or Alexa Fluor 488 Phalloidin containing DAPI (ThermoFisher Scientific) to enable accurate cell segmentation and zymosan particle counting. Images were acquired using the Leica TCS SP5 II confocal microscope and IN-Cell Analyzer 6,000 system with cell segmentation and particle counting performed using the IN-Cell Developer Toolbox v1.9. Phagocytic index was measured as follows:

Phagocytic Index = (No. of particles/cell) × (% of phagocytic cells).

For synaptosome uptake measurements, co-localization of pHrodo-red signal and CellMask Blue was performed using a custom MATLAB application and results expressed using the Pearson coefficient.

For measurements of phagocytic uptake in iMGL derived from HD and isogenic control lines, cells cultured for 30 days in 6-well plates were replated in 384-well poly-D-lysine and laminin-coated plates (Corning) at 5,000 cells per well and were allowed to rest at 37°C in 5% CO_2_ for 1 h. Separate wells were treated with 5 μM Cytochalasin D (an inhibitor of actin polymerization) or vehicle (complete medium). After 1 h, cells exposed to pHrodo^™^ Red Zymosan Bioparticles^™^ (0.5 μg/mL per well, ThermoFisher Scientific). Uptake of zymosan bioparticles was measured by the pHrodo^™^ fluorescent intensity per cell confluence in images acquired in Incucyte S3 (Sartorius).

### Tissue processing for CyTOF mass cytometry

Mice were anesthetized using isoflurane/O_2_ mix in an induction chamber (Dräger Fabius, 8604200-14). Absence of reactivity to pain was tested by tail and paw pinch followed by transcardial perfusion with 1X HBSS (10 U/mL heparin) using a peristaltic pump at a rate of 6 mL/min for 5 min. Forebrains and spleens were collected in 5 mL of ice-cold 1X HBSS and stored on ice until tissue collection was completed. Brains were dissociated using the Papain Dissociation System (Worthington) by two sequential triturations and incubation for 15 min at 37°C. Single cell suspensions were filtered through a 40 μm cell strainer in 50 mL Falcon tubes and resuspended in 30% Percoll in AutoMACS running buffer (Miltenyi Biotec) at room temperature (RT) and centrifuged for 15 min at 500 xg with no brake to generate a floating top layer of myelin. Following myelin removal, 1X AutoMACS running buffer was added to the remaining volume, any pelleted cells were resuspended. Cells were filtered again through 40 μm cell strainers and centrifuged at 300 xg for 10 min at RT to obtain an immune cell-enriched pellet. The experimental workflow is summarized in Supplementary Figure S2.

Spleens were homogenized by mechanical dissociation followed by red blood cell lysis using 1X RBC lysis buffer (ThermoFisher Scientific) for 10 min at RT per manufacturer’s recommendation. Following RBC lysis, samples were supplemented with 1X PBS, filtered through a 40 μm cell strainer and centrifuged at 300 xg for 10 min. Following a centrifugation at 300 xg for 5 min, a cocktail of 32 metal tag-conjugated antibodies (1:100) in Maxpar staining buffer was added to the cells and incubated for 1 h at 4° C. Information on antibody panel used for cytometry including clone and source is included in Supplementary Table S1. Several monoclonal antibodies were conjugated to metal tags in-house using the Maxpar 8X antibody labeling kit (Fluidigm) according to manufacturer’s protocol. All labelled antibodies were quality-controlled in-house for correct labeling prior to use.

Following immunostaining, cells were washed by three sequential centrifugations at 300 xg for 5 min at 4°C in Maxpar staining buffer followed by fixation with 4% PFA in 1X PBS overnight at 4°C.

### iPSC microglia surface staining for CyTOF mass cytometry

Cells were gently detached through medium exchange and washed once in Maxpar cell staining buffer. Following a centrifugation at 300 xg for 5 min a cocktail of 34 metal tag-conjugated antibodies (1:100; Supplementary Table S3) in Maxpar staining buffer was added to the cells and incubated for 1 h at 4°C. Following immunostaining, cells were washed by three sequential centrifugations at 300 xg for 5 min at 4°C in Maxpar staining buffer followed by fixation with 4% PFA in PBS overnight at 4°C.

### Mass cytometry data acquisition and analysis

Prior to data acquisition, fixed cells were incubated with Maxpar 191/193Ir DNA Intercalator (50 nM final concentration, Fluidigm) diluted in 4% PFA in PBS at 4°C overnight. Cells were washed twice in Maxpar H_2_O (Fluidigm) and centrifuged at 800 xg for 15 min at RT. Cells were resuspended in EQ Four Element Calibration beads (Fluidigm) diluted in Maxpar H_2_O (1:4), filtered through a 40 μm cell strainer to remove cellular aggregates and debris followed by data acquisition on a Helios mass cytometer (Fluidigm). The full sample volume was acquired at an event rate of maximum 500 events/s.

Mass cytometry data were analyzed using different computational tools available in Cytobank.^2^ Normalization beads were gated out and single live cells were then gated based on DNA content and cell length to exclude debris and doublets. Identification of immune cell subsets was then performed using traditional bivariate gating (Supplementary Figure S2). In brain, cell populations were defined based the following gating strategy: microglia/resident myeloid, CD45^low^CD11b^+^CX3CR1^+^; neutrophils, CD45^high^CD11b^+^Ly-6G^+^; monocytes/macrophages, CD45^high^CD11b^+^Ly-6G^−^; dendritic cells, CD45^high^CD11b^+^CD11c^+^; T cells, CD45^high^CD11b^low^CD3^+^. In spleen immune subsets were defined as follows: CD4 T helper cells: CD45^+^ CD3^+^CD4^+^CD8^−^; CD8 cytotoxic T cells: CD45^+^CD3^+^CD4^−^CD8^−^; B cells, CD45^+^CD19^+^CD3^−^; NK cells: CD45^+^CD335^+^; Neutrophils: CD45^+^CD3^−^CD19^−^CD11b^+^Ly-6G^+^; Monocytes/macrophages: CD45^+^CD3^−^CD19^−^CD11b^+^Ly-6G^−^; Dendritic cells: CD45^+^CD3^−^CD19^−^CD11b^+^CD11c^+^Ly-6G^−^; Mast cells: CD45^+^FceRIa^+^. Differentiated iMGL were identified as CD45^+^ cells. The percentage of each cell population was calculated out of the total of CD45^+^ cells. To visualize all live immune cells or all iMGL in a single two-dimensional map, we applied an unsupervised high-dimensional data analysis on concatenated fcs files pooling cells equally and randomly sampled from all mice or samples in each group, using the t-distributed stochastic linear embedding (t-SNE) algorithm available on Cytobank (viSNE algorithm).

### Synaptosome purification

Six-month-old Sprague Dawley rats were anesthetized with isoflurane and brains were dissected, placed in 10 volumes of ice cold HEPES-buffered sucrose (0.32 M sucrose, 4 mM HEPES pH 7.4) and homogenized using a Dounce homogenizer. Homogenate was spun at 1,000 xg at 4°C for 10 min to remove the pelleted nuclear fraction (P1). Supernatant was spun at 15,000 xg for 20 min to yield a crude synaptosomal pellet (P2), which was resuspended in 10 volumes of HEPES-buffered sucrose. After centrifugation at 10,000 xg for an additional 15 min at 4°C, the washed crude synaptosomal fraction (P2’) was layered in ultra-clear centrifuge tubes (Beckman) onto 4 mL of 1.2 M sucrose and centrifuged at 230,000 xg for 15 min (Beckman SW40Ti). The interphase was collected, layered onto 4 mL of 0.8 M sucrose and centrifuged at 230,000 xg for 15 min to yield the synaptosome pellet. Purified synaptosomes were conjugated with pHrodo^™^ Red succinimidyl ester (ThermoFisher Scientific, P36600) in 0.1 M sodium carbonate (pH 9.0) by incubation for 2 h at RT with gentle agitation. Unbound pHrodo^™^ dye was removed by multiple rounds of washing and centrifugation with 1X HBSS and pHrodo^™^-conjugated synaptosomes were then resuspended in 5% DMSO in 1X HBSS and stored at −80°C until further use.

### Immunoblotting

Using a cordless pestle motor for 1 min homogenization per sample (VWR), human and mouse brain tissues were homogenized on ice in 1X RIPA buffer (Sigma-Aldrich) containing protease and phosphatase inhibitors (Roche). BMDM and tissue lysates were centrifuged at 15,000 xg for 20 min at 4°C and protein concentration was assessed in the supernatant (BCA protein assay kit, ThermoFisher Scientific, 23,225, United States). Equal amounts of protein in Laemmli sample buffer (Biorad) were separated by gel electrophoresis using 4–12% Bis-Tris polyacrylamide gels (Invitrogen) and transferred to PVDF membranes (Merck Millipore). Nonspecific binding was blocked by incubating membranes for 1 h in TBS blocking buffer (Odyssey, Licor) followed by overnight incubation at 4°C with the following primary antibodies: anti-C1q (Abcam, ab11861, 1:500), anti-C3 (Abcam, ab97462, 1:250), anti-TSPO (Abcam, ab109497, 1:5,000), and anti-β-actin (Cell Signaling technology, 3700S/4970S, 1:5,000). Blots were washed three times in 1X TBS with 0.1% Tween-20 (TBS-T) followed by incubation for 1 h with fluorophore-conjugated secondary antibodies (Licor, IRDye 680LT Goat anti Mouse/Rabbit, 1:1,000) at RT. After three additional washes in TBS-T, blots were imaged using the Odyssey CLx Imaging system (Licor). ImageJ was used to quantify protein levels normalizing to actin levels as a loading control.

### Microglial phagocytosis in acute mouse brain sections

Microglial phagocytic activity in R6/2 mice and WT littermate control acute brain sections was assessed as previously described in Keaney et al. (2019). Briefly, 300 μm brain slices were generated from transgenic and litter mate controls following euthanasia and kept in pH and temperature-controlled conditions. Slices were then incubated with pHRodo-conjugated zymosan beads or synaptosomes for 1 h at 37°C and 4% CO2, non-phagocytosed beads & synaptosomes were removed by gentle washing of the tissue sections followed by fixation with 4% PFA for 1 h at RT. Fixed slices were incubated for 3 h with blocking solution (5% normal goat serum, 0.05% Triton-X in PBS) followed by incubation with anti-Iba1 (polyclonal Guinea Pig, dilution 1:500, Synaptic Systems, 234004) for 48 h. Slices were washed three times for 15 min in 1X PBS and incubated with secondary antibody (anti-guinea pig IgG, Alexa488; Thermo Fisher Scientific, 1:1,000) for 3 h at RT. After washing three times for 5 min in 1X PBS, slices were counterstained with DAPI (ThermoFisher Scientific, D1306) and mounted onto glass slides. Images were acquired using the Zeiss LSM880 confocal microscope. The number of particles engulfed by Iba1-positive microglia and microglia morphological features were quantified. Experimental workflow is summarized in Supplementary Figure S3.

### Microglia morphology analysis

Multiple z-stack images (20–30 stacked images, 8–10 μm thickness) of Iba1-positive microglia were captured from random areas of *ex vivo* acute brain slices using the Zeiss Axioscan and flattened using Zeiss ZEN 2.3 software. Microglial morphology analysis was performed using a customized morphology Fiji-script developed in collaboration with Prof. Winnok de Vos (University of Antwerp).

### Quantification of synaptic markers by mass spectrometry (LC–MS/MS)

Mouse and human brain tissues (Supplementary Table S2) or sections were homogenized (1-part tissue and 4-parts RIPA buffer) using an ultrasonicator (Covaris). Samples were sonicated three times and after each cycle of sonication, the sample were centrifuged at 1,000 xg for 5 min. The supernatant was collected, and remaining pellet was further homogenized, repeating the procedure twice. The supernatants collected from the two consecutive runs were centrifuged at 1,000 xg for 10 min to remove any cell debris. Twenty microliters of homogenate (~400 μg total protein) were reduced and alkylated using tris (2-carboxyethyl) phosphine (TCEP) and iodoacetamide. The protein was then precipitated by adding 6 volumes of cold acetone stepwise with vigorous mixing and incubated at −20°C for 2 h. After incubation samples were centrifuged at 20,000 xg for 15 min. The supernatant was discarded and the resulting pellet was washed with acetone:water (6:1 v/v). After the washing step, the pellet was air dried and suspended in 200 mM ammonium bicarbonate buffer containing trypsin at an enzyme substrate ratio of 1:10. Stable labelled peptide cocktail was added at this stage and the mixture was incubated overnight at 37°C in an Eppendorf thermomixer. After digestion the samples were diluted 1:1 with 95:5 water:acetonitrile containing 0.1% formic acid.

Data was acquired using AB Sciex QTRAP 6500 triple quadruple mass spectrometer coupled to a Schimadzu LC system. Chromatographic separation was achieved on an Aeris C18 peptide column (100 × 2.1 mm, 2.6 μm) running at 0.5 mL/min. Aqueous mobile phase consisted of water with 0.1% formic acid and organic mobile phase consisted of acetonitrile with 0.1% formic acid. The digested samples containing both endogenous and labelled peptides were analyzed by scheduled Multiple Reaction Monitoring (MRM) method monitoring 101 transitions over 30 min run time.

### Golgi-Cox staining, imaging, and quantitation of dendritic spines

Frozen mouse and human tissues were immersed in a 5 mL/1 cm^3^ tissue Golgi-Cox impregnation solution (FD Rapid GolgiStain^™^ Kit, FDNeuroTechnologies, INC). The solution was changed 24 h after the first immersion. After 2 weeks impregnation, samples were transferred into a washing solution (solution C) and stored at RT in the dark for at least 72 h. Sagittal sections (100 μm) were mounted on microscope slides, dried, and stained for 10 min before being dehydrated and cleared in xylene (QPS Neuropharmacology, Austria). Slides were imaged on a confocal microscope (63x/1.40 Oil DIC, Zeiss LSM 880) using the reflection mode, z-stacking (100 μm range, 0.5 μm interval) and tiling options. Neurons from the region of interest fulfilling the following criteria were imaged: (1) the presence of untruncated dendrites, (2) a consistent/dark impregnation along the entire extent of arborization, and (3) a relative isolation from neighboring stained neurons to avoid interference and ensure accuracy of dendritic spine counting. Striatal and middle frontal gyrus neurons were imaged in mice and human brains, respectively. Eight neurons per donor/animal were imaged. Once acquired, the 3D stacks were flattened into a 2D image. The 2D images were opened in ImageJ, each neurite length measured via the freehand line tool, and each spine counted manually. All tissue sample IDs were blinded for experimenters at all steps of the analysis and until completion of the study.

### Single nuclei transcriptome analysis

Single nuclei RNA sequencing dataset from post-mortem caudate and putamen tissue of HD and control donors deposited in NBCI GEO: GSE152058 (Lee et al., 2020) was analyzed with a main focus in microglia. Count matrices were loaded into the R (v4.1.0) package Seurat (v4.3.0) (Butler et al., 2018). SCTransform (v0.3.5) was used for count normalization with regressing out the percentage of mitochondria genes. The top principal components (PCs), determined by the ElbowPlot function, were selected for dimensionality reduction, clustering and visualization with UMAP. Marker genes for each cluster were identified with FindAllMarkers function in Seurat (v4.3.0) with the following criteria: adjusted *p*-value <0.05, log fold change ≥0.25, and genes detected in >25% of the cells within its corresponding cluster. Top markers of microglial subclusters were showed by a heatmap plot using DoHeatmap function in Seurat (v4.3.0). Pseudobulk differential expressed genes were shown in a volcano plot using EnhancedVolcano package (v1.12.0). Gene oncology (GO) analysis was performed in DAVID using differential expressed genes (Huang et al., 2009; Sherman et al., 2022). Significantly upregulated GO terms were shown by bar plots via GraphPad Prism 9 or GOPlot package (v1.0.2).

### Statistical analysis

All data are presented as means/individual values ± standard error of the mean (SEM). Data and statistical analysis were performed in GraphPad Prism. Mass cytometry data were analyzed using the unpaired Student’s *t*-test with discovery determined using the two-stage linear step-up procedure of Benjamini, Krieger and Yekutieli, and a False Discovery Rate (FDR; Q) of 10%. Unpaired two-tailed Student’s *t*-test was also used to analyze Western blot data. A Two-way ANOVA followed by Sidak’s multiple comparison was used for spine density and *ex vivo* phagocytosis assays. Linear/non-linear regressions were performed for body weight evolution and human microglial function over time.

## Results

### HD R6/2 mice display a progressive disease phenotype

The R6/2 mouse model has been extensively used as a preclinical model of HD and as such a wealth of information on its disease phenotypes and progression has been reported. This model displays progressive motor dysfunction, weight loss and brain atrophy in the cerebral cortex and the striatum (Stack et al., 2005). To ensure the disease progression of mice housed in our facilities closely resembles the reported phenotypes and progression we assessed body, brain and spleen weight longitudinally at 7, 10, or 15 weeks of age (Supplementary Figure S1). We confirmed a progressive drop in body weight starting at 7 weeks of age, with significant decreases compared to WT litter mates starting at 10 weeks of age (Supplementary Figure S1A). Reduction in the body weight was paralleled by visible and significant decreases in spleen and brain weight in R6/2 mice at pre-and post-symptomatic stages of disease (7–15 weeks of age; Supplementary Figures S1B–D). These findings confirmed R6/2 mice display gross alterations in immune and neurodegenerative phenotypes in line with previous reports (Lee et al., 2018).

### *Ex vivo* mass cytometry reveals phenotypic and functional hyperactivity in peripheral innate immunity of R6/2 mice

Mutant huntingtin can induce both autonomous and non-cell autonomous innate immune activation starting at pre-symptomatic stages of human disease. It is unclear whether heightened inflammatory responses are also present in the R6/2 model. A study from Pido-Lopez et al. described a hyperactive innate immune response in R6/2 mice and when blunted by chlodronate-induced depletion attenuated systemic inflammation (Pido-Lopez et al., 2018). Conversely, Lee et al. report that innate and adaptive immune responses in R6/2 mice are impaired and that stimulation with LPS/TLR4 pathway activation reverses these deficiencies resulting in beneficial behavior and survival outcomes (Lee et al., 2018). Beyond these contradictory findings, to date no phenotypic or functional characterization of peripheral innate and CNS immunity has been performed in the course of disease progression. As such, to get a holistic view on peripheral and CNS immune status in R6/2 mice we performed *ex vivo* mass cytometry studies of spleen and brain at 9 weeks (early symptomatic) and 13 weeks (late symptomatic disease) of age. A CyTOF mass cytometry panel of antibodies was assembled to define distinct immune cell populations and gain insights into their phenotypes.

**Figure 1:**
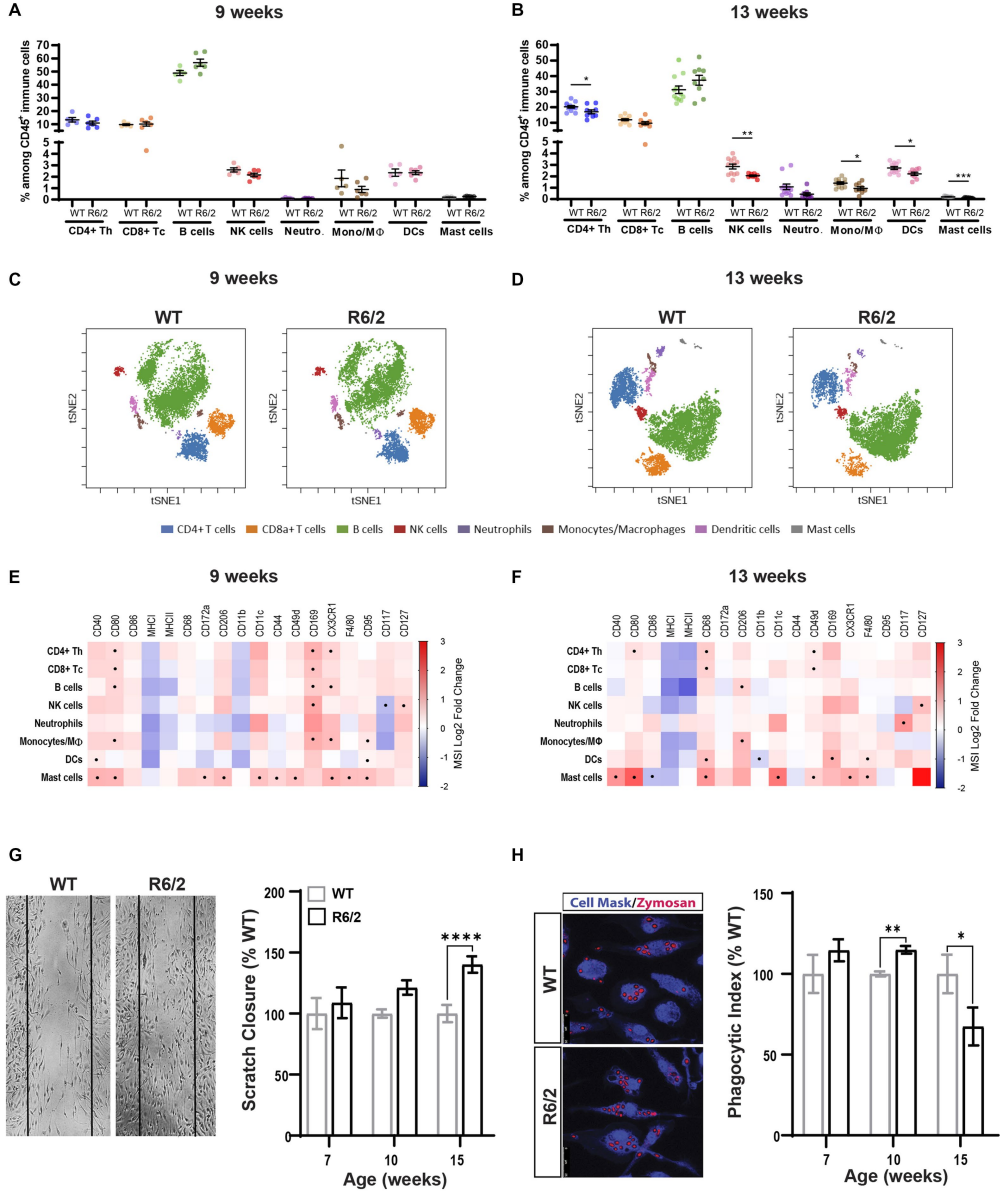
Immune phenotyping and functional characterization of peripheral immunity in R6/2 mice. **(A,B)** Relative abundance of the immune subsets among all CD45+ immune cells in the spleen compartment in 9-and 13-week-old mice. CD4+ Th: CD4 positive T helper lymphocyte; CD8+ Tc: CD8 positive T cytotoxic lymphocyte: B cells: B lymphocytes; NK cells: Natural killer cells; Neutro: Neutrophils; Mono/Mϴ: monocytes/macrophages; DCs: dendritic cells. **(C,D)** Representative two-dimensional projections of single-cell data generated by t-SNE of immune cell populations in WT vs. R6/2 mice at **(C)** 9 and **(D)** 13  weeks. **(E,F)** Heatmap representation summarizing levels of expression markers in immune cells populations at 9 and 13 weeks. Boxes marked with the symbol (•) show statistically significant genotype effect on marker expression (*p*-value equal to or less than 0.05). CRs: cytokine-related receptors. Data are presented as mean ± SEM. FDR with two-stage step-up method of Benjamini, Krieger and Yekutieli (*Q* = 10%); *p < 0.05, **p < 0.01, ****p < 0.0001. **(G)** Longitudinal assessment of migratory function in WT and R6/2 BMDM measured as scratch surface area closure due to cell invasion including representative image at 15  weeks of age. **(H)** Uptake of pHrodamine-conjugated zymosan particles in BMDMD isolated from femurs of aging WT and R6/2 mice including representative image at 10 weeks of age. Data represents mean values ± SEM. Statistical significance was evaluated using Two Way ANOVA, Sidák multiple comparison test, *p < 0.05, **p < 0.01, ****p < 0.0001; ns, not significant. *N* = 4–5 animals.

Mass cytometry analysis of spleens revealed no changes in cell numbers across innate and adaptive cell populations in the spleen at 9 weeks of age (Figures 1A,C) and a decrease in the CD4^+^ T helper cells as well as the innate immune subsets including NK, monocyte, dendritic and mast cells in the R6/2 mice at 13 weeks of age (Figures 1B,D). Phenotypic characterization revealed significant increases in regulators of phagocytic activity (CD68, CD206), cytoskeletal rearrangement (migratory and homing signals, CD11b, CD11c, CD44, CD49d), and antigen presentation (CD8+ T cell cross-priming: CD40, CD80, F4/80, CD127, CD169) at pre-symptomatic and to a lesser degree at the symptomatic stages of disease. These data collectively suggest a heightened inflammatory response prior to symptom onset and likely cell exhaustion and death at the late stages of disease (Figures 1E,F).

To further corroborate the functional implications of these phenotypic observations we generated bone morrow-derived macrophage (BMDM) cultures for WT and R6/2 mice at timepoints pre-and post-phenotypic characterization. Cells were first tested for any alterations in their ability to invade the surface area of the mechanical scratch in the cell culture wells over a 24 h period. Small and non-significant increases of migratory activity of R6/2 BMDM were measured at ages of 7 and 10 weeks reaching statistical significance at 15 weeks of age (Figure 1G). Next, we assessed phagocytic functions by exposing WT and R6/2 BMDM to pHRodo Zymosan^™^ bioparticles (which fluoresce when internalized into acidified endocytic compartments) for 24 h. A significant increase of phagocytic activity in the R6/2 mouse BMDMs at 10 weeks of age was measured aligning with earlier phenotypic observations (Figure 1H). A significant decrease in phagocytosis was observed at 15 weeks of age, likely due to overall decrease in cellular fitness at end stages of disease.

### Immune phenotyping of R6/2 brain tissue reveals altered microglia activation states and peripheral immune cell influx to the CNS compartment

**Figure 2:**
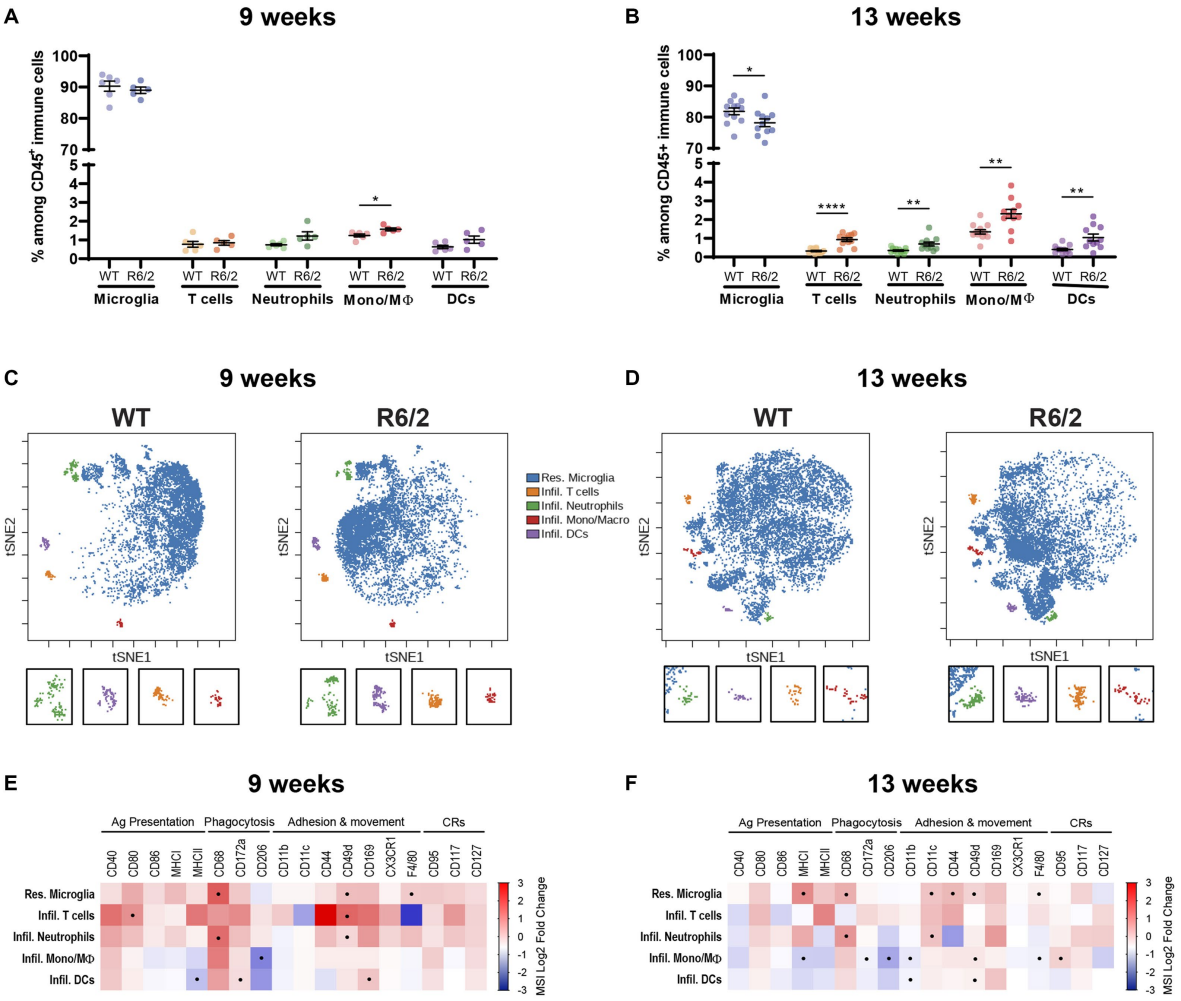
Characterization of the brain’s resident and infiltrating immune cells populations by mass cytometry. **(A,B)** Relative abundance of the immune subsets among all CD45+ immune cells in the brain compartment in 9-and 13-week-old mice. Mono/Mϴ: monocytes/macrophages; DCs: dendritic cells. **(C,D)** Representative two-dimensional projections of single-cell data generated by t-SNE of immune cell populations in WT vs. R6/2 mice at 9 and 13  weeks. **(E,F)** Heatmap representation summarizing levels of expression markers in immune cells populations at **(E)** 9 and **(F)** 13 weeks. Boxes marked with the symbol (•) show statistically significant alterations in marker expression. CRs: cytokine-related receptors. *N* = 6 (9  weeks) – 11 (13  weeks) animals/condition. Data are presented as mean ± SEM; FDR with two-stage step-up method of Benjamini, Krieger and Yekutieli (*Q* = 10%); *p < 0.05, **p < 0.01, ****p < 0.0001.

To determine whether the aberrant peripheral inflammatory tone was paralleled in microglia we performed mass cytometry of the brain homogenates collected from the same cohort of WT and R6/2 mice. Microglial activation states and peripheral immune cell presence/infiltration were assessed in parallel. At 9 weeks of age there were no significant changes in microglia cell numbers and a slight increase in mononuclear phagocyte numbers (perivascular, choroid plexus or infiltrating macrophages). At 13 weeks microglial numbers decreased accompanied by increasing cellular heterogeneity due to infiltration of peripheral myeloid and lymphoid immune subsets in the brain of R6/2 mice (Figures 2A,B). Assessment of cell surface marker levels in t-SNE revealed a phenotypic shift in microglia in both early and late stages of disease for the R6/2 mice (Figures 2C,D). At pre-symptomatic stages microglia (and infiltrating neutrophilic populations) increased inflammatory markers such as CD68 and F4/80. With disease progression additional markers including MHCI, CD11c and CD44 were increased in both monocytes and microglia further supporting a phenotypic shift toward a pro-inflammatory state (Figures 2E,F).

### Microglial heterogeneity increases with disease progression in R6/2 mice

**Figure 3:**
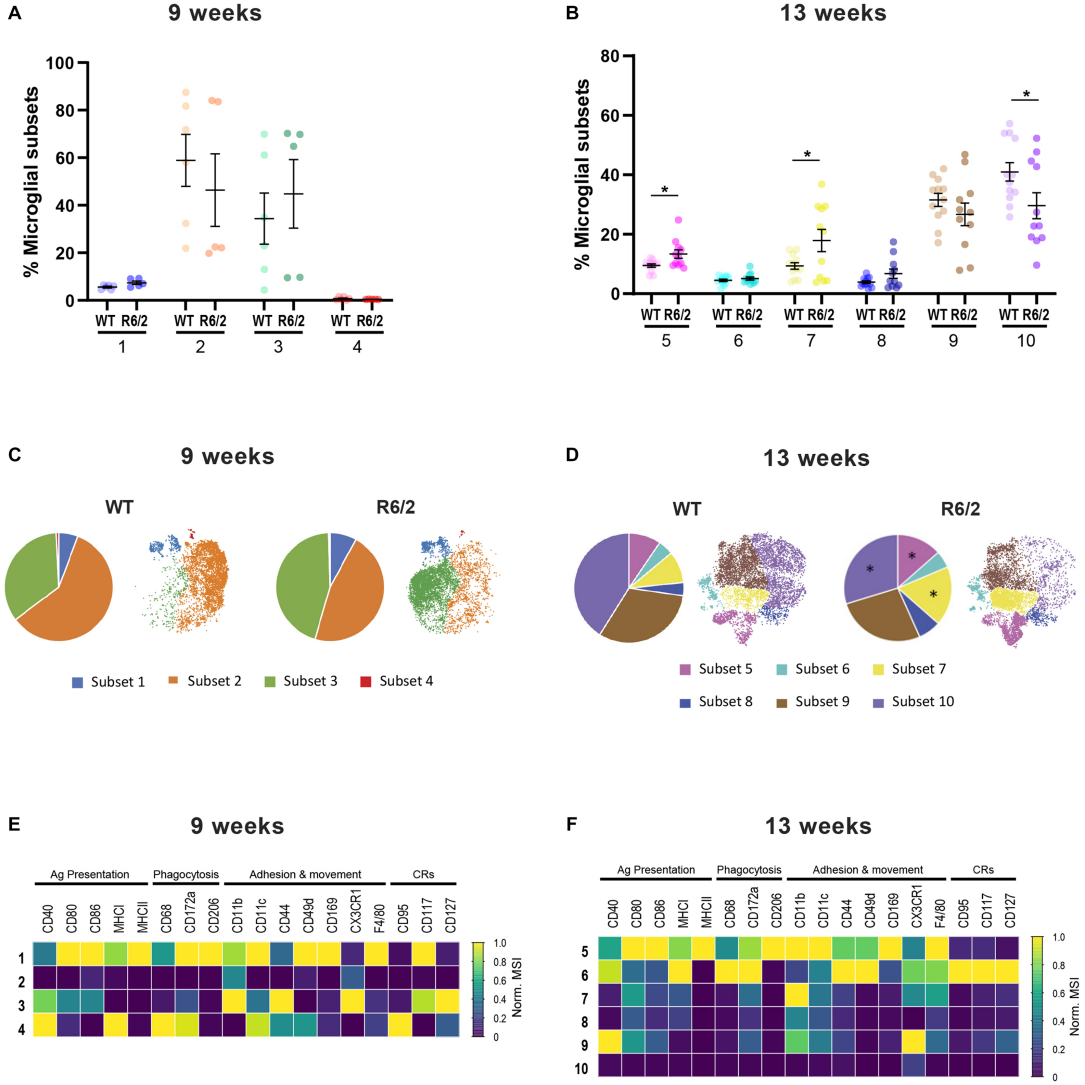
Characterization by mass cytometry of microglia heterogeneity in R6/2 mice. **(A,B)** Relative abundance of the microglia subsets among total population in the brain compartment in 9-and 13-week-old mice. **(C,D)** Pie charts and viSNE clustering showing the representation of microglial subsets in WT and R6/2 mice at 9 and 13 weeks. **(E,F)** Heatmap capturing levels of expression markers in microglia subsets at 9 and 13 weeks. CRs: cytokine-related receptors. *N* = 6 (9 weeks) – 11 (13 weeks) animals/condition; data are presented as mean ± SEM; FDR with two-stage step-up method of Benjamini, Krieger and Yekutieli (*Q* = 10%); *p < 0.05.

Next, we performed in depth profiling of microglial populations (CX3CR1^+^/CD45^low/med^) to further investigate whether a shift to enhanced inflammatory responses was characteristic of all brain microglia. Mass cytometry data from WT and R6/2 mice were mapped in viSNE revealed four clusters of microglia in R6/2 and WT mice at 9 weeks of age (Figure 3A). R6/2 microglia in clusters 1 and 4 displayed increased antigen presentation, phagocytic and migration markers. Interestingly, the increase in these subsets countered a decrease in microglia cluster 2 lacking all activation markers (potentially homeostatic; Figures 3A,C,E). An increase of microglial phenotypic heterogeneity was observed with age and disease progression to advanced stages. At 13 weeks of age six microglial clusters distinct from those in week 9 were identified. Cluster 5 and 7 microglia, characterized by elevated levels of co-stimulatory molecules, phagocytic and migratory markers were significantly increased in R6/2 mice, while cluster 10 microglia lacking any of the above-mentioned markers (homeostatic) were significantly decreased (Figures 3B,D,F).

### R6/2 Microglia are morphologically distinct and display heightened phagocytic synaptic uptake

Following characterization of microglial populations, we examined the impact of the observed phenotypic alterations on microglial function. Since migration/surveillance and phagocytic regulators were increased in R6/2 microglia we assessed microglial morphology and phagocytic uptake in a complex 3-dimensional environment consisting of acute 300 μm brain slices pre-and post-phenotypic characterization time points. Brain sections generated from 7-, 10-, and 15-week-old mice were exposed to synaptosomes conjugated to pHRodamine. Synaptosomes were utilized to compare propensities for synapse recognition and uptake between WT and R6/2 microglia during disease progression. One-hour post-zymosan or synaptosome exposure brain sections were washed, fixed in PFA and stained for Iba1. Images of acute sections were analyzed for microglial morphology (surrogate for migratory activity) and phagocytic uptake of pHrodo^+^ particles by Iba1-positive cells, excluding pHRodamine signal from phagocytic non-microglial cells (i.e., astrocytes). Although this particular assay does not capture synaptic pruning by microglia in real time, it provides insights on the functional states of the microglia and alternations in propensity of synapse recognition and uptake in a 3 dimensional native and complex environment.

**Figure 4:**
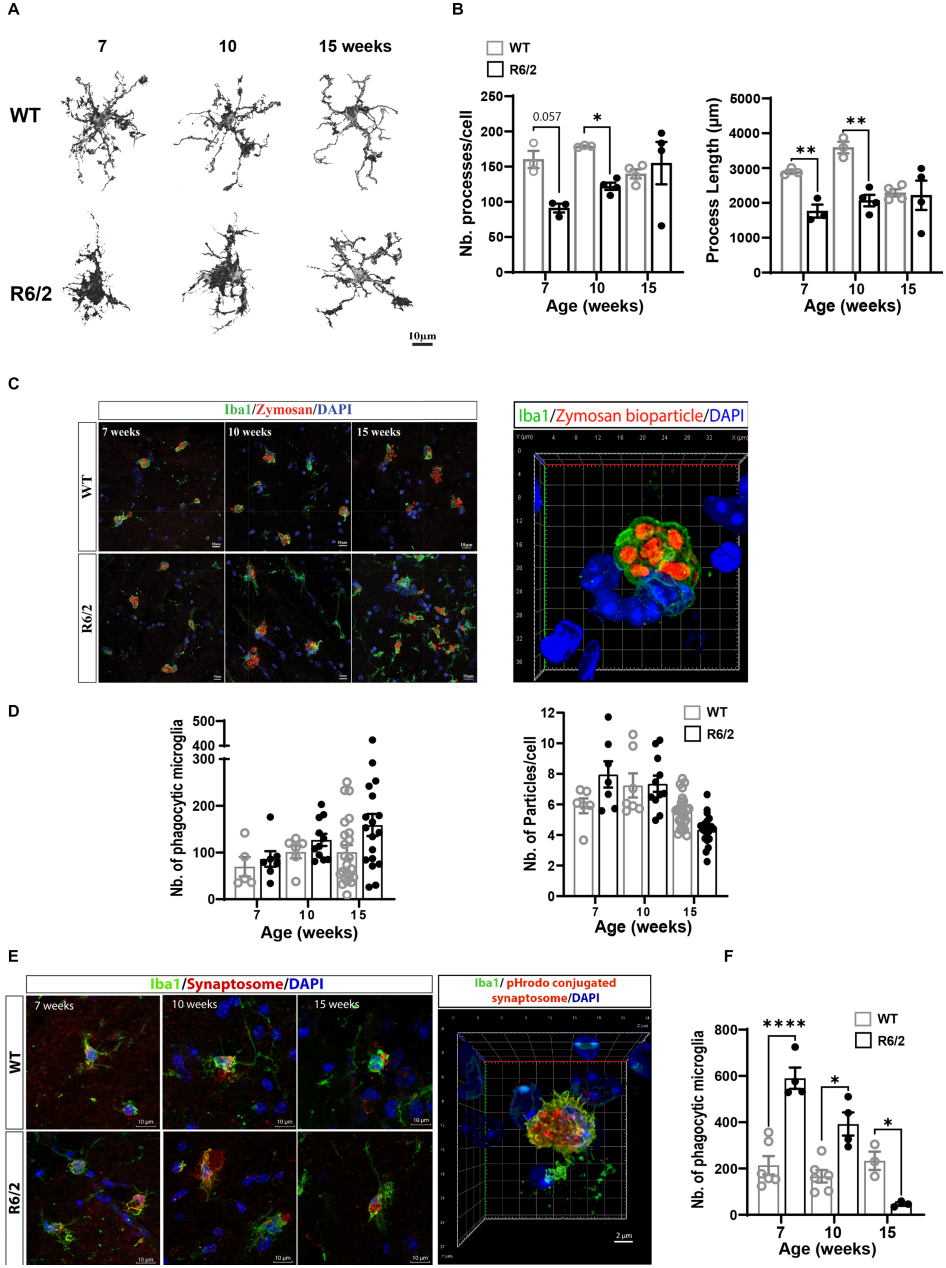
*Ex vivo* microglial morphology and phagocytic function in aging R6/2 mice. **(A)** Microglia morphological changes with age in WT and R6/2 mice. **(B)** Comparative quantitation of microglia filopodial branching during disease progression in R62 vs. age-and litter-matched WT mice. **(C)** Representative 3D-reconstructed image of a phagocytic microglia labeled with Iba1 (green; nuclei DAPI blue) displaying internalized pHrodo^™^ conjugated zymosan bioparticles^™^ (red). **(D)** Longitudinal assessment of microglial phagocytosis of zymosan bioparticles in aging WT and R6/2 mice (*n* = 5–23 brain slices/condition, *N* = 3–4 animals/condition). **(E)** Representative images of *ex vivo* microglial stained with Iba1 (green; nuclei DAPI blue) phagocytosis of pHrodamine-conjugated synaptosomes (red). **(F)** Comparative quantitation of the number of synaptosomes-phagocytosing microglia in aging WT and R6/2 mice (*n* = 3–6 brain slices/condition, *N* = 2–4 animals/condition. Statistical significance was evaluated using Two-Way ANOVA, Sidák multiple comparison test, *p < 0.05, **p < 0.01, ****p < 0.0001; ns, not significant).

Images and morphological analysis of microglia revealed a significant decrease in microglial ramifications measured by number of processes and process length in 10-week-old and 7-and 10-week-old R6/2 mice, respectively. At these ages R6/2 microglia displayed enlarged soma and shorter less abundant filopodial processes indicative of increased phagocytic function and motility (Figures 4A,B). Functional assessment of zymosan engulfment (macro-phagocytosis) showed non-significant differences in phagocytosed particle numbers and in numbers of phagocytic microglia at 7 and 10 and 15 weeks of age (Figures 4C,D). Next, we questioned whether R6/2 microglia showed an increased propensity for synaptic uptake. Acute exposure of R6/2 and WT brain slices to pHrodamine-conjugated synaptosomes showed significant uptake by R6/2 microglia at 7 and 10 weeks of age (Figures 4E,F). A significant decrease in synaptosome uptake was observed at the end stages of the disease (week 15; Figure 4F), potentially reflecting a microglial state characterized by an overwhelming cellular debris burden due to progressive neurodegeneration at the end stages of disease and a “shift in priorities” from synaptic pruning to overall phagocytic debris clearance.

### R6/2 mice display complement dysregulation, microglial activation, and reduced synaptic density

**Figure 5:**
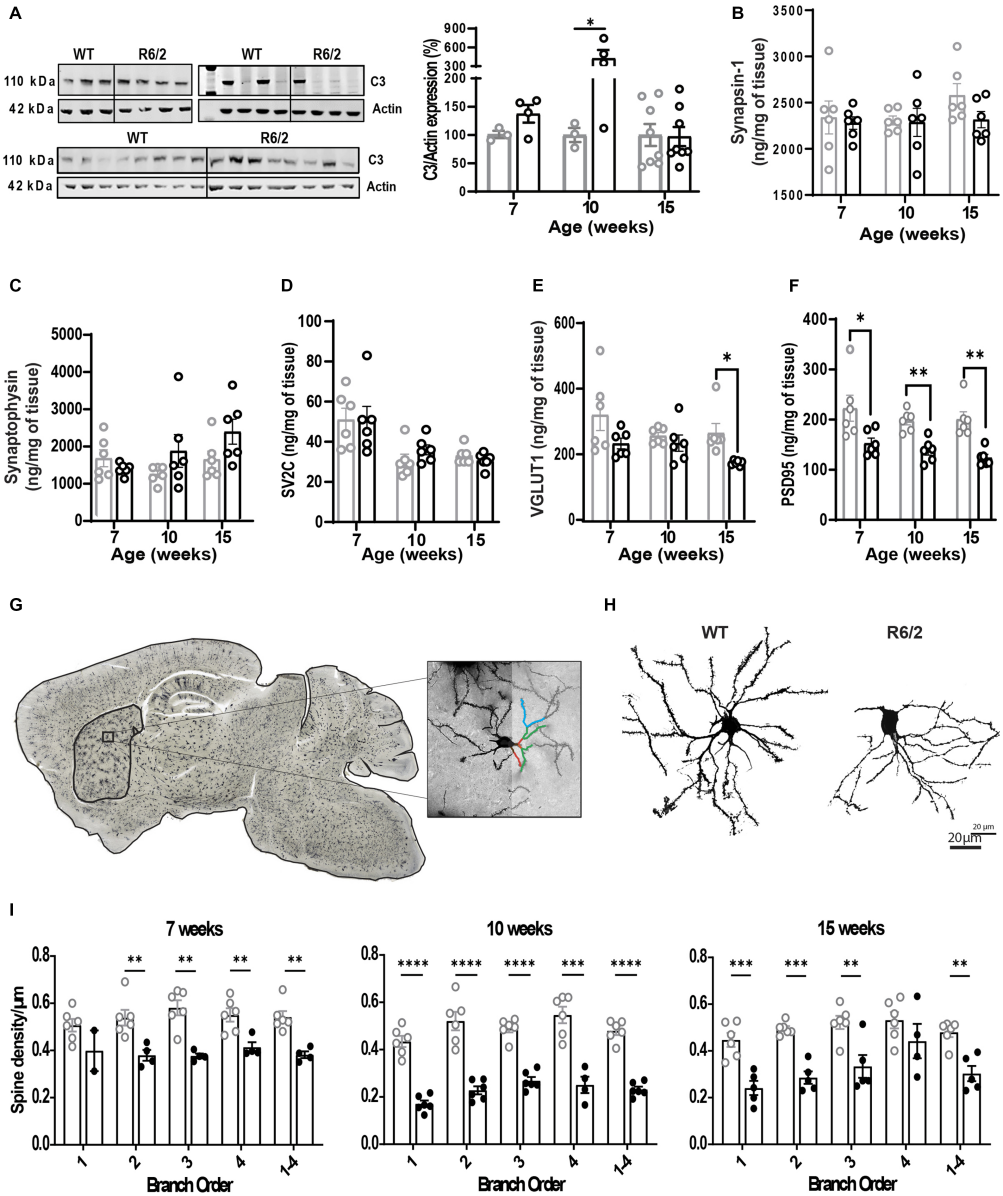
Altered synaptic architecture in aging R6/2 mice. **(A)** Representative image and densitometry analysis of complement factor C3 in WT and R6/2 brain homogenates at 7, 10, and 15 weeks of age normalized to actin. Quantitative measurements by mass spectrometry of pre-synaptic proteins synapsin-1 **(B)**, synaptophysin **(C)**, SV2C **(D)**, VGLUT1 **(E)** and post-synaptic protein PSD95 **(F)** in brain homogenates of WT and R6/2 mice at 7,10, and 15 weeks of age. **(G)** Bright field confocal image of a Golgi-Cox stained R6/2 brains, with a visual illustration of the manual neurite and dendritic spine tracing in striatal neurons. **(H)** High resolution bright field image of WT and R6/2 striatal neurons at 10 weeks of age. **(I)** Manual quantification of neurite branching and dendritic spine density in the striatal neurons of WT and R6/2 mice at 7, 10, and 15 weeks of age. *N* = 6 mice/condition; *n* = 8 neurons/mouse. Data shown as mean/individual values ± SEM. Analysis utilizing Two Way ANOVA, Sidák multiple comparison test, *p < 0.05, **p < 0.01, ***p < 0.001, ****p < 0.0001. ns, not significant.

Next, we questioned whether the observed enhanced microglial synaptic uptake would be reflected in reduced spine density in the aging R6/2 mice. To this end we assessed expression levels of complement factor C3 implicated in synaptic uptake and pre- (VGLUT1/2, synapsin, synaptophysin, SV2A/C) and post-synaptic markers (PSD95). Dendritic spine imaging of Golgi stained forebrains was also performed to assess spine density and overall arborization in R6/2 mice. C3 levels were significantly increased at 10 weeks of age but not at pre-symptomatic or late stages of disease (Figure 5A). No changes were observed in levels of pre-synaptic markers except for VGLUT1 at the terminal stages of disease (Figures 5B–E). In contrast a significant reduction of post-synaptic marker PSD95 was noted as early as pre-symptomatic stages of disease (7 weeks of age) and throughout disease (Figure 5F). Decreased levels of PSD95 were paralleled by significant loss of spines in primary through quaternary branching of striatal neurons in R6/2 mice (Figures 5G–I).

**Figure 6:**
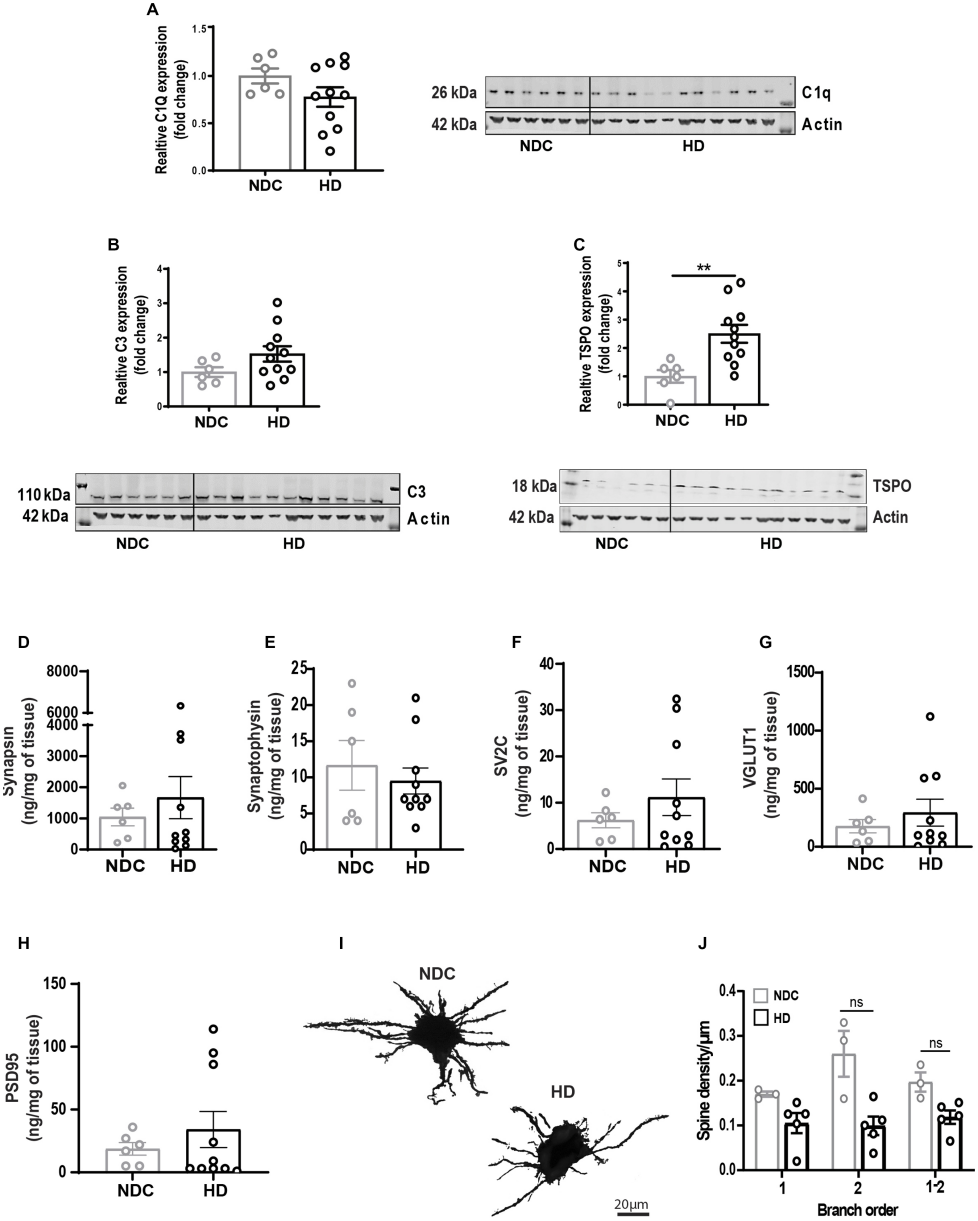
State of synaptic architecture in human HD brains. **(A–C)** Densitometry analysis of complement factors C1Q, C3, and glial activation marker TSPO western blots of human non demented controls (NDC) and HD brain homogenates (normalized to actin). Longitudinal evaluation by mass cytometry of pre-synaptic markers synapsin **(D)**, synaptophysin **(E)**, SV2c **(F)**, VGLUT1 **(G)** and post-synaptic marker PSD-95 **(H)** in human NDC and HD brain homogenates. **(I,J)** High magnification bright field confocal image of Golgi-Cox-stained middle frontal gyrus neurons and manual quantification of neurite branching and dendritic spine density in human NDC and HD. Data represented as mean/individual values ±SEM. *N* = 3–5 donors/condition; *n* = 8 neurons/donor. Statistical analysis was performed using Unpaired *t*-test (western blots) and Two Way ANOVA, Sidák multiple comparison test, **p < 0.01. ns, not significant.

Next, we questioned whether the state of synaptic architecture observed in R6/2 was reflected in human disease. Densitometry analysis of complement factors C1Q and C3 normalized to actin showed no significant changes in complement levels (Figures 6A,B); However, significant increase of translocator protein TSPO (Figure 6C) as a measure of inflammatory microglia was observed. Mass spectrometry analysis for the same pre-and post-synaptic markers showed no differences between non-demented controls (NDC) and HD brains (Supplementary Table S2; Figures 6C–H). Golgi-Cox staining of middle-frontal gyrus neurons showed a non-significant decrease in synaptic density in secondary branching of HD striatal neurons (Figures 6I,J).

### Microglia activation is characterized by upregulation of regulators of phagocytic and migratory functions in HD brain single cell sequencing data sets

**Figure 7:**
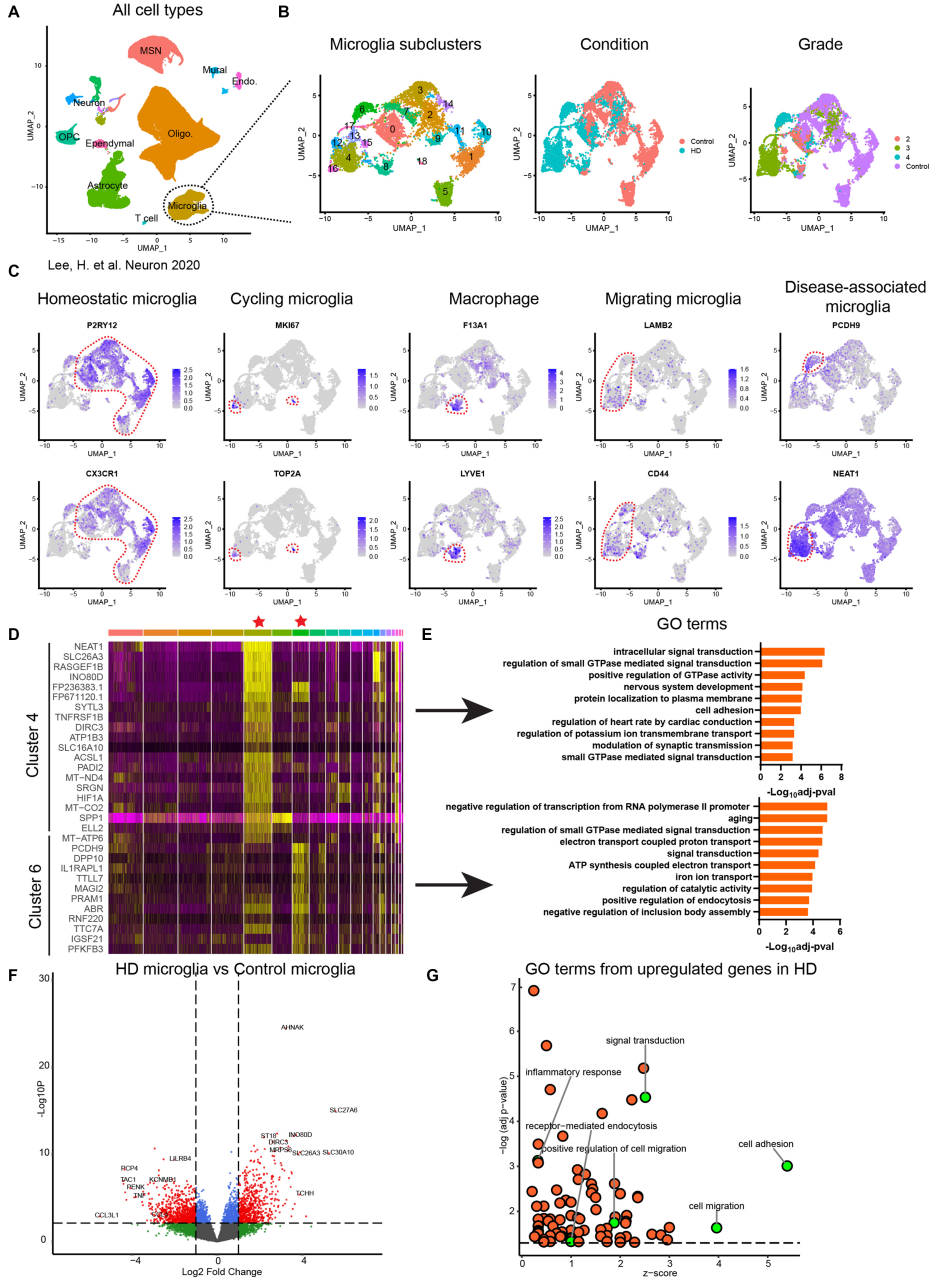
Transcriptomic characterization of human HD primary microglia. **(A)** Uniform Manifold Approximation and Projection (UMAP) of single nuclei transcriptome from published data set (Lee et al., 2020) comprising 125,467 nuclei from 14 unaffected control and 14 HD caudate and putamen samples. MSN: medium spiny neuron; Oligo.: oligodendrocyte; OPC: oligodendrocyte progenitor cell; Endo.: endothelial cell. **(B)** Subcluster identification corresponding to disease associated microglia. **(C)** Identification of microglia subpopulations based on the top markers of each cluster. **(D)** Heatmap of differential expressed genes in cluster 4 and cluster 6 compared to other clusters. **(E)** Enriched gene oncology (GO) terms of cluster 4 and cluster 6 based on upregulated genes in each cluster. **(F)** Differential gene expression analysis of whole microglia population without considering subclusters. Dash line indicates *p* = 0.05. **(G)** GO analysis based on the differential expressed genes in **(F)**. Dash line indicates *p* = 0.05.

To get a broader view of microglial population heterogeneity and key pathways dysregulated in disease-associated microglia we re-analyzed single nuclei RNA sequencing public datasets generated and reported by Lee et al. (2020). Analysis of datasets from 14 HD and 14 age-matched non-demented controls identified distinct cell populations including microglia, astrocytes, oligodendrocytes, OPCs, endothelial cells, ependymal cells, medium spiny neurons (MSN) and other neuron subtypes (Figure 7A). Further clustering of microglia resulted in 19 subclusters of microglia (Figure 7B) which suggests high level of heterogeneity of microglial populations. Five microglia clusters (1, 5, 9, 10, 11) unique to NDCs and 7 disease-specific microglia clusters (4, 6, 12, 13, 15, 16, 17) were identified (Figure 7B). Furthermore, when disease staging was considered as an analysis criterium, data showed certain microglia subsets cluster in a grade stage dependent manner, with clusters 4, 12, 13, 15 originating from brains at stage 3 of the disease and cluster 6 mainly from stage 4 (Figure 7B).

To further reveal the identity of these microglia subclusters, we performed differential gene expression analysis and found the majority of the microglia are in homeostatic stage which expressed high levels of CX3CR1 and P2RY12 (Figures 7C,D). A population of cycling microglia as defined by the expression of proliferation markers MKI67 and TOP2A and a cluster of macrophages defined by the expression of F13A1 and LYVE1 were identified (Figure 7C) (Hammond et al., 2019). Gene ontology analysis of disease-associated microglia in clusters 4 and 6 (representative of stage 3 and 4, respectively) was performed with the upregulated genes from each cluster. Distinct pathway enrichments in microglia from stage 3 and stage 4 brains were observed. Interestingly, cell migration, endocytosis, cell adhesion, signal transduction and inflammatory response were significantly upregulated in HD brains and were highly prioritized by the gene ontology analysis (Figures 7D–G).

### Human HD iMGL are characterized by highly phagocytic and migratory phenotypes and functions

**Figure 8:**
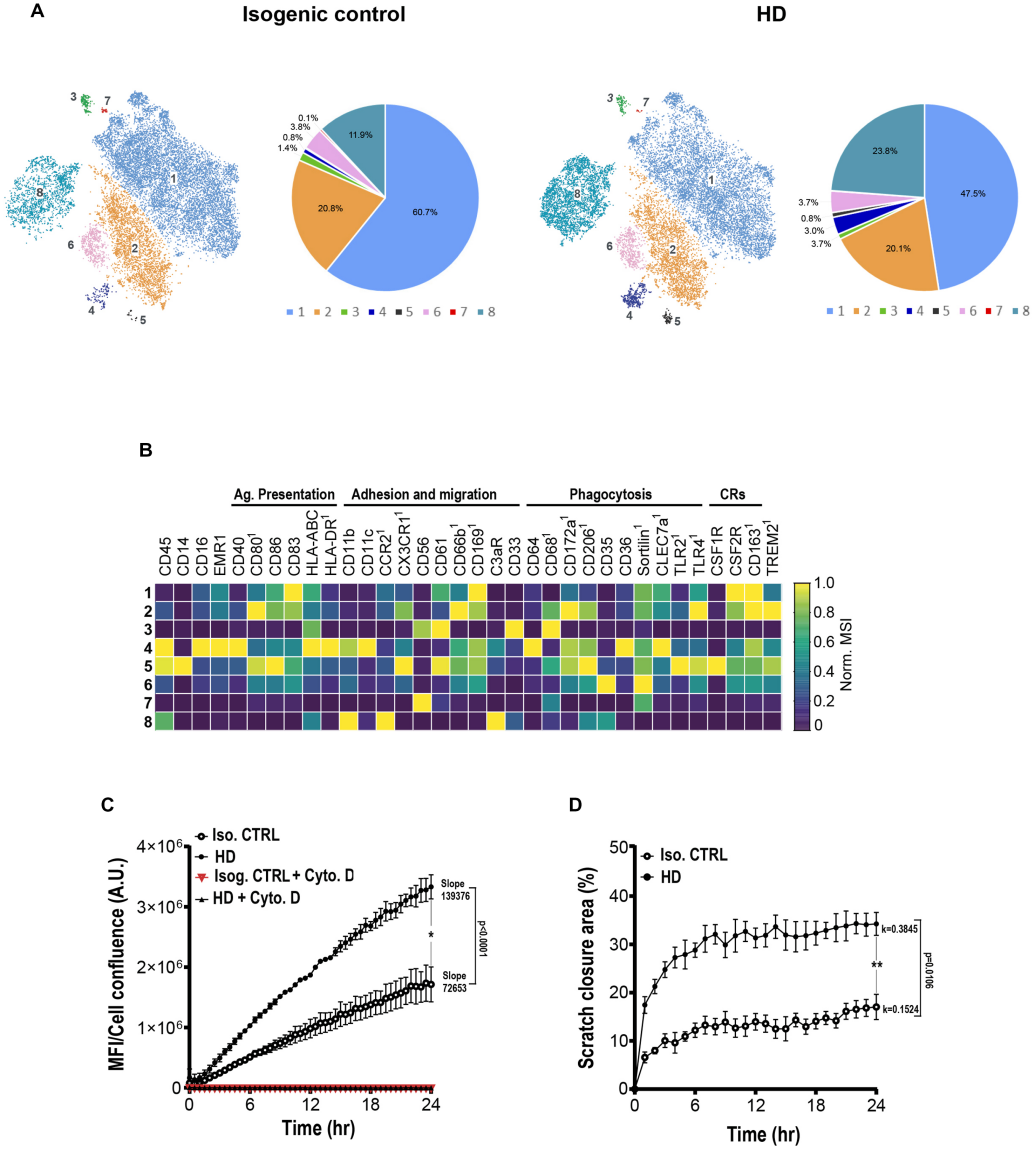
HD iPSC-derived microglia show enhanced phagocytosis and migration. **(A)** Representative mass cytometry t-SNE clustering and subset representation of human isogenic and HD iMGL. 15,000 cells per condition were randomly sampled. **(B)** Heatmap capturing levels of lineage, phagocytosis, migration and cytokine receptor cell markers in each microglial cluster. ^1^Marker showing low mean signal intensity (MSI < 5). **(C)** Graphic capturing longitudinal time lapse imaging over 24 h of pHrodo^™^ labelled zymosan uptake by isogenic and HD patient iMGL. Data includes blockade of phagocytosis with actin cytoskeleton inhibitor cytochalasin D. Fluorescence intensity was normalized to cell confluence. **(D)** Graphic representation of time lapse imaging of isogenic and HD iMGL migration to the scratch area over 24 h. Relative wound cellular density represents the proportion of migrating cells over time. Data from *N* = 3 independent iMGL differentiation experiments and *n* = 3 technical replicates. A linear **(C)** and nonlinear/exponential **(D)** regression were performed to evaluate longitudinal variations in phagocytosis/migration and compare slopes differences, and an unpaired *t*-test at 24 h to compare two means, *p < 0.05, **p < 0.01. Data captured as mean  ±S.D.

Next, we questioned whether increases in gene signatures regulating phagocytic and migratory pathways in human HD microglia signified overactive phagocytic and migratory phenotypes and functions. An HD patient iPSC line heterozygous for pathologic CAG repeats in the HTT gene (~18 repeats in one allele, and ~40 repeats in the other) and its isogenic control were induced to differentiate into iMGL. Following 25 days in culture, iMGL were detached and immunostained with a panel of metal-conjugated antibodies against cell surface regulators of essential microglial functions such as adhesion/migration, antigen presentation, and phagocytosis (Supplementary Table S3). High dimensional analysis at single cell resolution revealed 8 microglial sub-clusters, suggesting cells at day 25 in culture represent various states of maturity and activation (Figure 8A). A decrease in representation of cluster 1 characterized by high expression of tolerogenic receptors including CD83, CD163, CD169, and CSFR2 was observed in the HD line. This was paralleled by increased representation of clusters 4 and 5, characterized by high expression of adhesion, migration, and phagocytosis/lysosomal clearance receptors. Interestingly, representation of cluster 8 displaying high levels of peripheral innate immune markers such as CD45, CCR2, and complement receptor 3 (CR3) typically identifying cells of hematopoietic/monocytic lineage was increased in the HD line. This suggests a potential delayed maturity or propensity of HD stem cell subset to differentiate into monocytic lineage (Figures 8A,B).

Next, we assessed whether these phenotypic shifts observed by mass cytometry matched phagocytic and functional outcomes. Longitudinal functional assessment of phagocytic function by time lapse imaging showed both isogenic and HD lines were able to phagocytose and completely suppress phagocytic uptake in the presence of cytochalasin D (actin cytoskeleton polymerization inhibitor). A linear (Figure 8C) and nonlinear/exponential (Figure 8D) regression were performed to evaluate longitudinal variations in phagocytosis/migration and compare slopes differences, and a *t*-test at 24 h to compare two means. HD iMGL showed significantly enhanced particle uptake of pHRhodamine zymosan bioparticles with significant differences in slopes (*p* < 0.0001) and at the 24 h timepoint (*p* = 0.01; Figure 8C). Increased phagocytic activity was paralleled by a significantly increased migratory activity measured as scratch area invasion/closure by mHTT iMGL (k coefficient difference *p* = 0.0106; mean difference at 24 h *p* = 0.0028; Figure 8D). Interestingly, culture fluids collected form the same zymosan-stimulated microglia showed significantly higher levels of pro-inflammatory cytokine release (IL-6) by the HD cells compared to their isogenic controls, supporting their increased propensity for enhanced inflammatory response compared to isogenic controls (data not shown).

## Discussion

It is widely recognized that microglia may contribute to non-cell autonomous neurotoxicity and exacerbation of disease progression in various neurodegenerative diseases. Transcriptomics studies in animal models and more recently human brain tissues, have reported mHTT-and neuronal death-dependent microglial activation; however, what “activation” signifies and what cellular functions are ultimately impacted by deregulated gene transcripts have not been fully explored (Crotti et al., 2014). This study aimed at filling some of these gaps by establishing connections between microglial phenotypic alterations, functional behaviors, and impact on neuronal pathology in the R6/2 mouse model, human diseased tissues, and cellular systems. One outstanding question that remained given the decreases in spleen size with disease progression was: What phenotypic and functional changes occurred in the periphery pre-and post-symptomatic stages? The data regarding innate immune compartment to date is unclear with reports suggesting either deficiency or hyperactivation of immune response in HD rodent models (Lee et al., 2018; Pido-Lopez et al., 2018). Mass cytometry of the spleens and functional assessment of BMDMs showed an increase in regulators of phagocytosis and chemotaxis at pre-symptomatic stages followed by altered functional outcomes at symptomatic and end stages of disease. It is worth noting that *ex vivo* mass cytometry revealed presence of classical peripheral immune cell markers in the brains of R6/2 mice. We annotated these subsets as monocytes, neutrophils or DCs cells based on a combination of 3–4 markers present in the CyTOF panel and taking into consideration levels of CD45 expression in each subset. Due to the pathological inflammatory context and similar genetic makeup, we cannot exclude the possibility that these cells can also represent a subset of activated microglia, perivascular macrophages, or meningeal and choroid plexus innate immune cells. Peripheral immune cells have also been reported in HD human brain tissue; however, their implication in HD pathobiology requires additional investigation (Di Pardo et al., 2013).

The primary focus of our study was the microglia. More specifically we investigated how specific functions related to synaptic pruning such as phagocytosis and neuronal surveillance (morphology/chemotaxis) were impacted, considering the phenotypic alterations in these pathways. Immune phenotyping at single cell resolution demonstrated the presence of various microglial subsets in the R6/2 mice. This heterogeneity can be likely attributed to regionally-and metabolically-distinct microglial phenotypes and in the context of disease proximity to neuronal degeneration. Furthermore, basal ganglia are densely populated by microglia potentially contributing to heightened selective loss of striatal neurons in HD (Mittelbronn et al., 2001; Tan et al., 2020).

We assessed phagocytic activity in the entire surface areas of the brain slices and observed increased phagocytic activity of synaptosomes in the R6/2 microglia as early as pre-symptomatic stages of disease. The acute slice model was chosen to keep microglia in their complex environment omitting the caveats of cellular sorting and plastic-induced activation in functional assessment of brain isolated microglia (Cadiz et al., 2022). It is worth noting the differences in assay sensitivity of zymosan uptake vs. synaptosomes, with the synaptosome uptake being a more relevant and sensitive measure of alterations in phagocytosis. Assessment of R6/2 microglial functions required both assays given their more subtle phenotypes compared to HD iPSC microglia which displayed ameboid rather than ramified morphology *in vitro*. Synaptosome uptake by iMGLs was unquantifiable due to overly saturated signal *in vitro*. As such we resorted to a less sensitive approach such as zymosan uptake which showed significantly increased uptake in HD iMGL compared to isogenic control. Cytokine measurements in the culture fluids of iMGL exposed to zymosan showed an increased propensity for IL-6 and IL-8 release (data not shown). We recognize that one should not draw generalized conclusions for microglia function in HD based on hyperactivity observed in one donor line. Several donor lines with higher numbers of poly-Q repeats were also tested; however, no functional data were generated due to very poor overall health and viability of these cells when differentiated to iMGLs. Our observations with the high polyQ repeat lines align with others reporting significantly higher apoptotic activity in iMGL lines carrying over 80 polyQ repeats compared to those in the 30–40 range (O’Regan et al., 2021). Our iMGL *in vitro* data suggests mHTT can trigger an inflammatory phenotype in these cells independent of any non-cell autonomous activation cues from dying neurons in the complex brain environment.

In conclusion, our data from R6/2 mice, human cell and tissue-based systems collectively point to an autonomous and non-cell autonomous induction of innate immune inflammation, aberrant microglial migratory/surveillance and phagocytic functions associated with profound synaptic loss. It is broadly recognized that mHTT can cause multi-system toxicity that contributes to poor prognosis; however, in the context of neurological symptoms a microglial/innate immune modulator selectively targeting microglial aberrant functions such as synaptic overpruning may hold potential in attenuating progression of cognitive and motor decline in HD.

## Data availability statement

The datasets presented in this study can be found in online repositories. The names of the repository/repositories and accession number(s) can be found in the article/Supplementary material.

## Ethics statement

The studies involving human participants were reviewed and approved by UCB Biobank and Ethics Committee of UCB Biopharma. The patients/participants provided their written informed consent to participate in this study.

## Author contributions

JG and IK designed the studies. GG, JV, JK, AK, WF, and JG performed experiments and analyzed the data. JG and IK wrote the manuscript. All authors contributed to the article and approved the submitted version.

## Conflict of interest

The authors declare that this study was funded in its entirety by UCB Biopharma SRL. The funder had the following involvement in the study: all authors were employees of UCB at the time of execution of the study. The funder was involved in the funding of the study in its entirety, as well as study design, collection, analysis, interpretation of data, the writing of this article, and the decision to submit it for publication.

## Publisher’s note

All claims expressed in this article are solely those of the authors and do not necessarily represent those of their affiliated organizations, or those of the publisher, the editors and the reviewers. Any product that may be evaluated in this article, or claim that may be made by its manufacturer, is not guaranteed or endorsed by the publisher.

## Glossary

**Table 1:** 

BBB	Blood brain barrier
BMDM	Bone marrow derived macrophages
DAM	Disease-associated microglia
CNS	Central nervous system
CSF1R	Colony stimulating factor 1 receptor
CyTOF	Mass cytometry by time of flight
FDR	False discovery rate
HD	Huntington’s disease
HTT	Huntingtin protein
iHPC	Induced hematopoietic progenitor cells
iMGL	hiPSC-induced microglia
iPSC	Induced pluripotent stem cells
PFA	Paraformaldehyde
PLX3397	Pexidartinib
PolyQ	Polyglutamine
snRNAseq	Single nucleus RNA sequencing
TCEP	Tris (2-carboxyethyl)phosphine
Tg	Transgenic
t-SNE	t-distributed stochastic neighbor embedding
VEGF	Vascular endothelial growth factor
WT	Wild type

## Footnotes

^1^www.brainbank.nl

^2^https://cytobank.org
