# Supplementary material for: Innate immune activation and aberrant function in the R6/2 mouse model and Huntington’s disease iPSC-derived microglia
Source: Front Mol Neurosci. 2023 Jun 20;16:1191324. doi: 10.3389/fnmol.2023.1191324 (PMC10319581; doi:10.3389/fnmol.2023.1191324)
Supplement: Supplementary file 1 [file Data_Sheet_1.docx]

Supplementary Material

Innate immune activation and aberrant phagocytic function in the R6/2 mouse model and Huntington’s disease iPSC-derived microglia

**Gasser J^1, 2^**^*^**, Gillet G^1, 2^, Valadas J S^1, 2^, Rouvière L^1, 2^, Apoorva Kotian^3^, Wenqiang Fan^1,2^, Keaney J^1,2^, Kadiu I^1,2*^**

^1^Neuroinflammation Focus Area, ^2^Neuroscience Therapeutic Area, ^3^Development science, Early Solutions, UCB Biopharma SRL, Chemin du Foriest, 1420 Braine l’Alleud, Belgium

***Correspondence:**Julien.Gasser@ucb.com; Irena.Kadiu@ucb.com

**Figure S1**


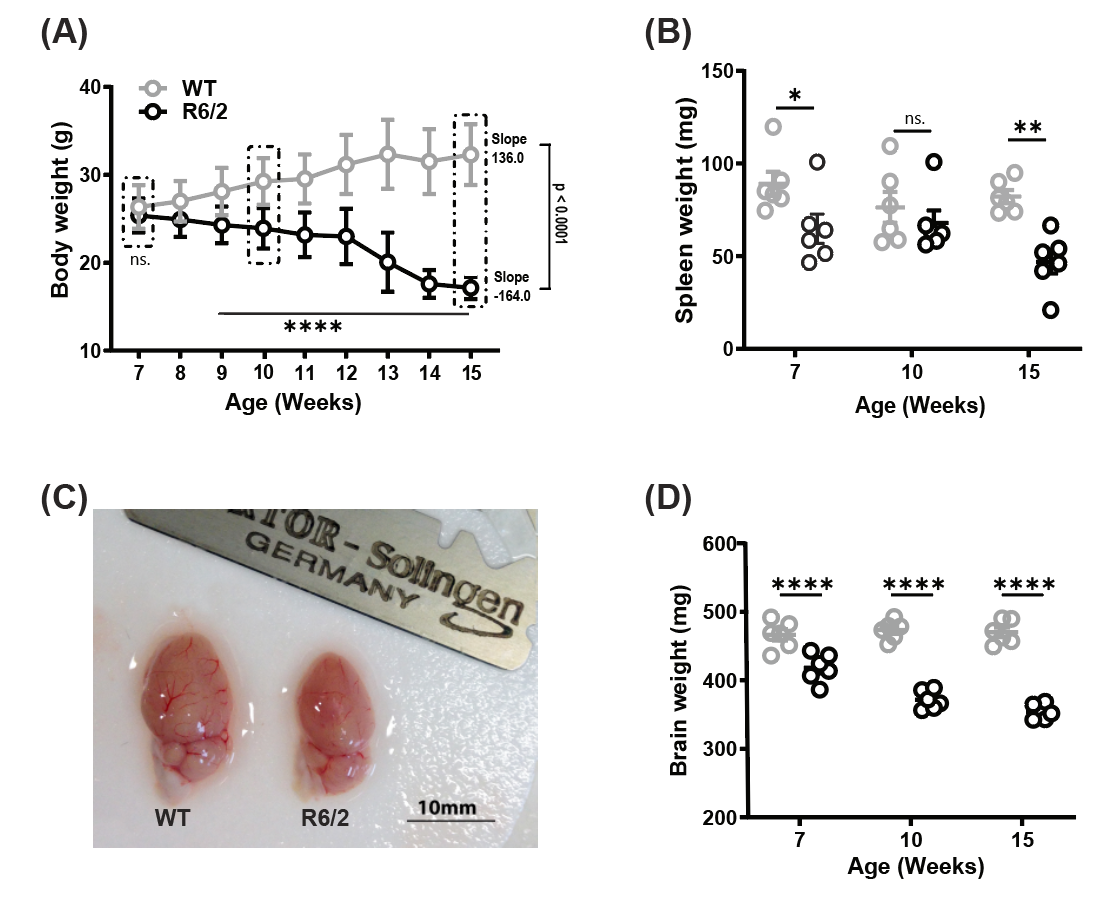


**Supplemental Figure 1. HD R6/2 mice show a progressive reduction of body, brain, and spleen weight.** (A) Weekly body weight decline in aging R6/2 mice vs age matched litter mate controls. (B) Declining spleen weight in R6/2 mice of 7, 10 and 15 weeks of age. (C) Representative image of brains from WT and R6/2 mice demonstrating brain atrophy at 15 weeks of age. (D) Significant reduction of brain weight at pre- and post-symptomatic stages of disease in R6/2 mice compared to WT controls. Data represents individual values ± SEM. N = 6 animals/condition. Two-way ANOVA, Sidák multiple comparison test, **p < 0.01; ****p < 0.0001. A linear regression was performed to evaluate longitudinal variations in body weight and compare slopes differences, *: p<0.05, **: p<0.01, ****: p<0.0001.

**Figure S2**


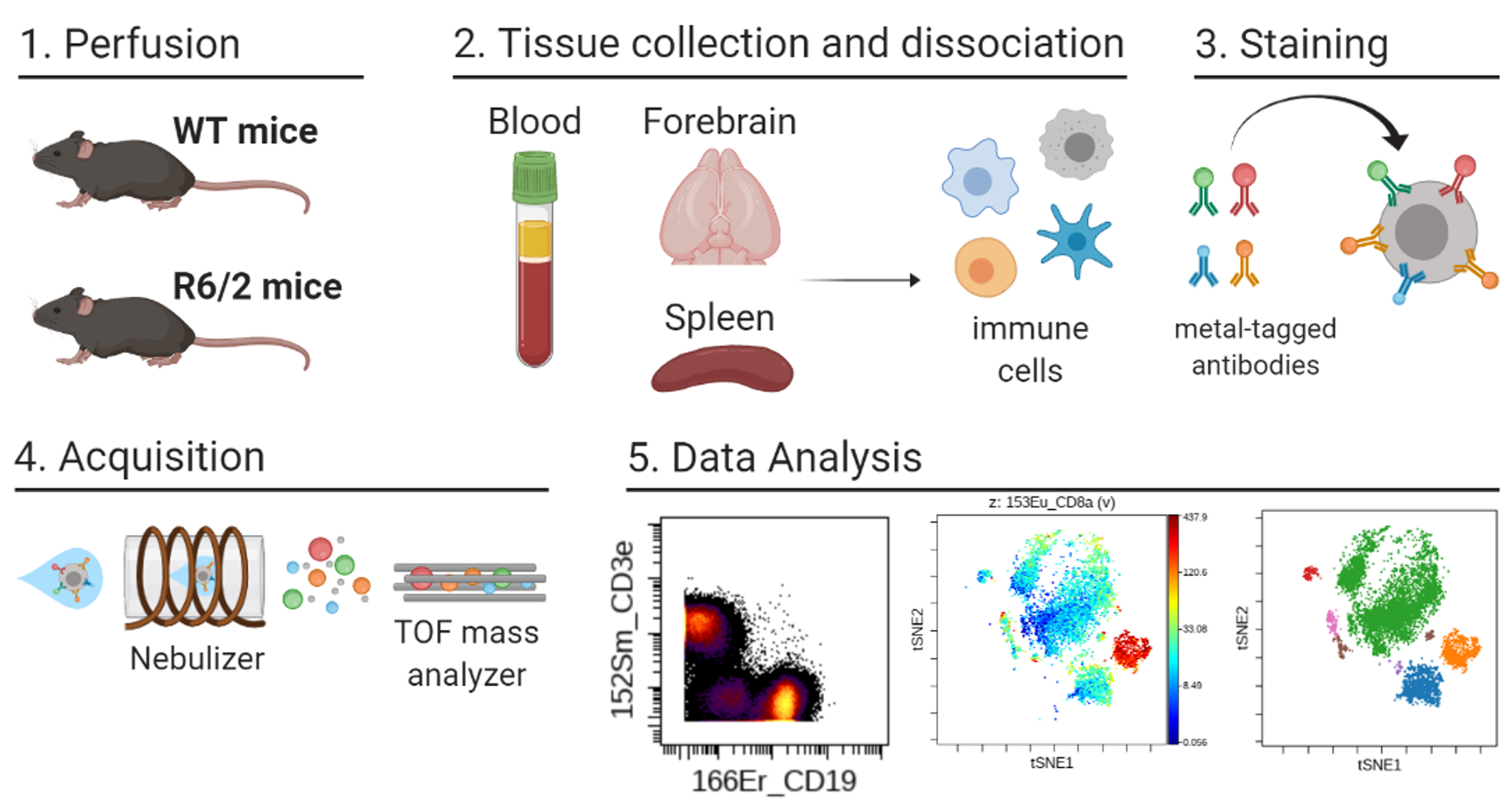


**Supplemental Figure 2. Schematic representation of the experimental workflow for mass cytometry (CyToF) immunophenotyping.** (1-2) Immune cells were isolated from brain, spleen and peripheral blood of 9 and 13-week-old R6/2 and WT mice. (3) Single-cell suspensions were stained with a cocktail of metal-tagged antibodies. Antibody panel is displayed in Supplemental Table 1. (4) Samples were analyzed using a Helios mass cytometer. Figure created in BioRender.

**Figure S3**


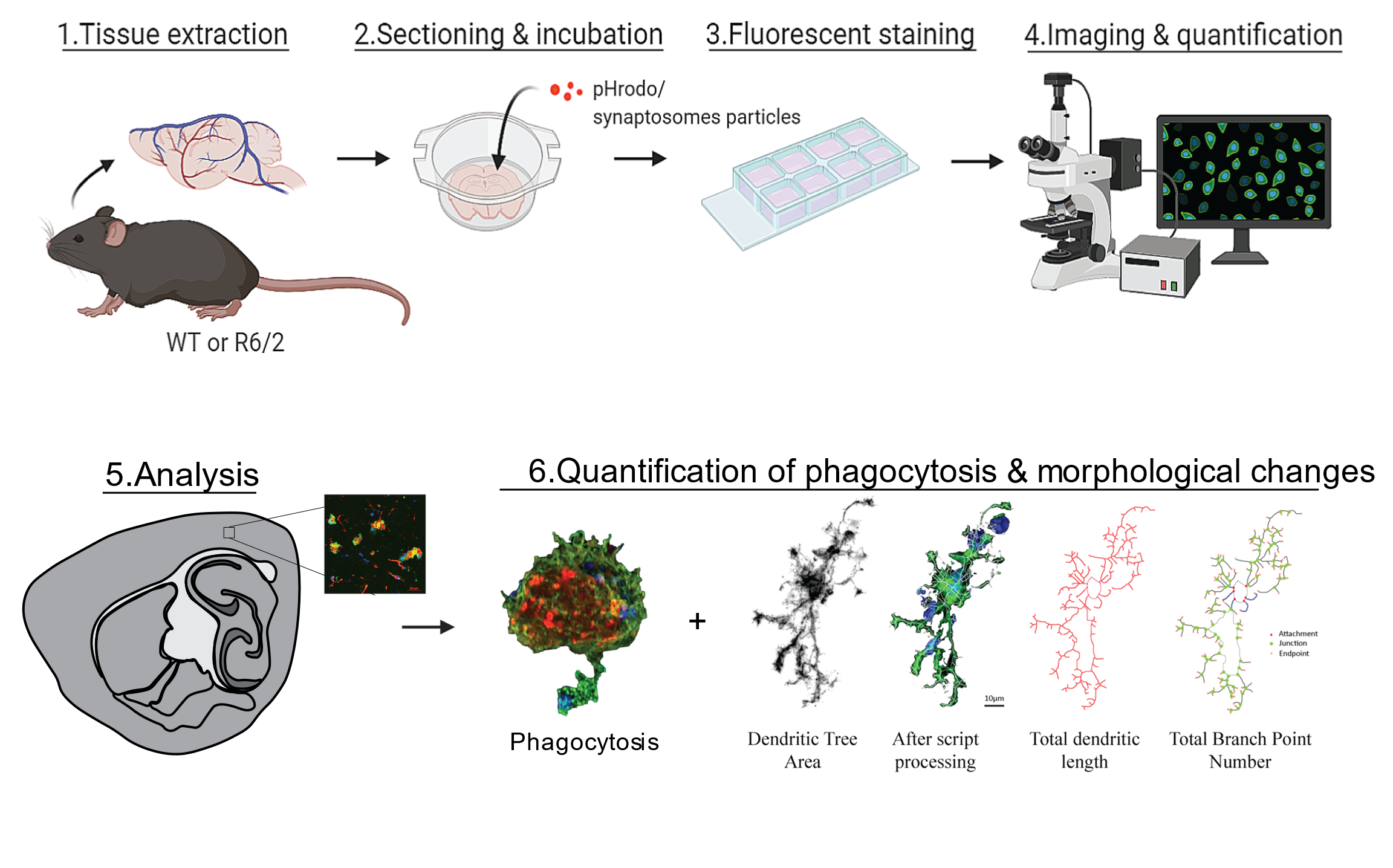


**Supplemental Figure 3. Schematic representation of the experimental workflow for ex vivo functional assessment of microglial biology.** (1-2) 300µm acute slices cells were generated form freshly extracted mouse brain. (3) After 1 hour incubation the slices were fed with pHRodo^TM^-conjugated zymosan bioparticles for an additional hour before being fixated with 4% paraformaldehyde. (4) Once fixed and immunostained, the slices were imaged using a Zeiss LSM 880 confocal microscope. (5-6) Microglial phagocytosis and morphology were then imaged, and phagocytosis quantified manually. Microglial morphology analysis was performed using a custom-made morphology Fiji-script. Figure created in BioRender.

| **UniProt** | **Isotope Tag** | **Antigen** | **Clone** | **Supplier** | **Catalog Number** |
| --- | --- | --- | --- | --- | --- |
| P35461 | 141Pr | Ly-6G | 1A8 | Fluidigm | 3141008B |
| P97797 | 142Nd | CD172a | P84 | Biolegend | 144002 |
| P01899 | 144Nd | MHCI | 28-14-8 | Fluidigm | 3144016B |
| P06332 | 145Nd | CD4 | RM4-5 | Fluidigm | 3145002B |
| Q61549 | 146Nd | F4/80 | BM8 | Fluidigm | 3146008B |
| P06800 | 147Sm | CD45 | 30-F11 | Fluidigm | 3175010B |
| P05555 | 148Nd | CD11b | M1/70 | Fluidigm | 3148003B |
| P31996 | 149Sm | CD68 | Fa-11 | Biolegend | 137002 |
| P15379 | 150Nd | CD44 | IM7 | Fluidigm | 3150018B |
| Q00651 | 151Eu | CD49d | R1-2 | Fluidigm | 3151016B |
| P22646 | 152Sm | CD3e | 145-2c11 | Fluidigm | 3152004B |
| P01731 | 153Eu | CD8a | 53-6.7 | Fluidigm | 3153012B |
| Q9ES52 | 154Sm | TER119 | TER-119 | Fluidigm | 3154005B |
| Q6QLQ4 | 155Gd | CLEC7a (CD369) | RH1 | Biolegend | 144302 |
| P70658 | 159Tb | CXCR4 | L276F12 | Fluidigm | 3159030B |
| P27512 | 161Dy | CD40 | HM40-3 | Fluidigm | 3161020B |
| Q9QXH4 | 162Dy | CD11c | N418 | Fluidigm | 3162017B |
| P25446 | 163Dy | CD95 (FAS) | SA367H8 | Biolegend | 152602 |
| Q9Z0D9 | 164Dy | CX3CR1 | SA011F11 | Fluidigm | 3164023B |
| Q08481 | 165Ho | CD31 | 390 | Fluidigm | 3165013B |
| P25918 | 166Er | CD19 | 6D5 | Fluidigm | 3166015B |
| Q8C567 | 167Er | CD335 | 29A1.4 | Fluidigm | 3167008B |
| P09581 | 168Er | CSF1R (CD115) | AFS98 | Biolegend | 135521 |
| Q2HZ94 | 169Tm | CD206 (MMR) | C068C2 | Fluidigm | 3169021B |
| Q62230 | 170Er | CD169 | 3D6.112 | Fluidigm | 3170018B |
| Q00609 | 171Yb | CD80 (B7-1) | 16-10A1 | Fluidigm | 3171008B |
| P42082 | 172Yb | CD86 (B7-2) | GL1 | Fluidigm | 3172016B |
| P05532 | 173Yb | c-kit (CD117) | 2B8 | Fluidigm | 3173004B |
| Q61790 | 174Yb | CD223 (LAG3) | C9B7W | Fluidigm | 3174019B |
| Q60943 | 175Lu | CD127 (IL7Ra) | A7R34 | Fluidigm | 3175006B |
| P20489 | 176Yb | FcεRIa | Mar | Fluidigm | 3176006B |
| P04441 | 209Bi | I-A/I-E (MHCII) | M5/114.15.2 | Fluidigm | 3209006B |

**Supplemental Table 1. Detailed antibody panel used for CyToF.** The full metal-conjugated antibody panel used for CyToF mass cytometry staining of both brain and spleen samples. Antibodies were used at 1:100 in Maxpar staining buffer. Cells were stained in 100µL final staining volume.

**Supplemental Table 2:** Clinical information on Non demented control (NDC) and Huntington’s disease (HD) brain tissue donors.

| **UniProt** | **Isotope tag** | **Antigen** | **Clone** | **Supplier** | **Order ID** |
| --- | --- | --- | --- | --- | --- |
| P06800 | 89Y | CD45 | HI30 | Fluidigm | 3089003B |
| O19689 | 141Pr | HLA-A, B, C | W6/32 | Fluidigm | 3141010B |
| P15509 | 143Nd | GM-CSFR/CSF2R/CD116 | 31916 | R&D Systems | MAB706 |
| Q01151 | 144Nd | CD83 | HB15 | Biolegend | 305302 |
| Q14246 | 145Nd | EMR1 | A10 | Biorad | MCA2674 |
| P12314 | 146Nd | CD64 | 10.1 | Fluidigm | 3146006B |
| P20702 | 147Sm | CD11c | Bu15 | Fluidigm | 3147008B |
| P08637 | 148Nd | CD16 | 3G8 | Fluidigm | 3148004B |
| Q99523 | 149Sm | Sortilin | 334703 | R&D Systems | MAB31541-100 |
| Q9BZZ2 | 150Nd | CD169 | 7-239 | Biolegend | 346002 |
| Q9BXN2 | 151Eu | CLEC7a (Dectin-1) | 15E2 | Biolegend | 355402 |
| P16671 | 152Sm | CD36 | 5-271 | Fluidigm | 3152007B |
| P41597 | 153Eu | CD192 (CCR2) | K036C2 | Fluidigm | 3153023B |
| Q86VB7 | 154Sm | CD163 | GHI/61 | Fluidigm | 3154007B |
| P13591 | 155Gd | CD56 (NCAM) | B159 | Fluidigm | 3155008B |
| P42081 | 156Gd | CD86 | IT2.2 | Fluidigm | 3156008B |
| O00206 | 158Gd | CD284 (TLR4) | HTA125 | Fluidigm | 3158024B |
| P07333 | 159Tb | M-CSFR/CSF1R/CD115 | 61708 | R&D Systems | MAB329 |
| P33681 | 161Dy | CD80 | 2D10.4 | Fluidigm | 3161023B |
| P31997 | 162Dy | CD66b | 80H3 | Fluidigm | 3162023B |
| P78324 | 163Dy | CD172a/b (SIRPa/b) | SE5A5 | Fluidigm | 3163017B |
| Q9NZC2 | 164Dy | TREM2 | 237920 | R&D Systems | MAB17291-100 |
| P25942 | 165Ho | CD40 | 5C3 | Fluidigm | 3165005B |
| P17927 | 166Er | CR1/CD35 | E11 | Biolegend | 333402 |
| P11215 | 167Er | CD11b | ICRF44 | Fluidigm | 3167011B |
| P22897 | 168Er | CD206 (MMR) | 15.2 | Fluidigm | 3168008B |
| P20138 | 169Tm | CD33 | WM53 | Fluidigm | 3169010B |
| Q16581 | 170Er | C3aR | hC3aRZ8 | Biolegend | 345802 |
| P34810 | 171Yb | CD68 | Y1/82A | Fluidigm | 3171011B |
| P49238 | 172Yb | CX3CR1 | 2A9-1 | Fluidigm | 3172017B |
| O19762 | 173Yb | HLA-DR | L243 | Fluidigm | 3173005B |
| P08571 | 175Lu | CD14 | M5E2 | Fluidigm | 3175015B |
| O60603 | 176Yb | CD282 (TLR2) | TL2.1 | Fluidigm | 3176021B |
| P05106 | 209Bi | CD61 | VI-PL2 | Fluidigm | 3209001B |

**Supplemental Table 3. Detailed antibody panel used for CyToF study of iPSC microglia.** Antibodies were used at 1:100 in Maxpar cell staining buffer, in 100µL final volume.
